# The Specificity of ParR Binding Determines the Incompatibility of Conjugative Plasmids in Clostridium perfringens

**DOI:** 10.1128/mbio.01356-22

**Published:** 2022-06-21

**Authors:** Thomas D. Watts, Daouda A. K. Traore, Sarah C. Atkinson, Carmen Lao, Natalie Caltabiano, Julian I. Rood, Vicki Adams

**Affiliations:** a Infection Program, Monash Biomedicine Discovery Institute and Department of Microbiology, Monash University, Victoria, Australia; b Department of Biochemistry and Molecular Biology, Monash University, Victoria, Australia; c Faculté des Sciences et Techniques, Université des Sciences, des Techniques et des Technologies de Bamako (USTTB), Bamako, Mali; Yale University School of Medicine

**Keywords:** plasmid partitioning, plasmid maintenance, plasmid incompatibility, *Clostridium perfringens*, ParR, *parC*, surface plasmon resonance, DNA binding, analytical ultracentrifugation

## Abstract

Plasmids that encode the same replication machinery are generally unable to coexist in the same bacterial cell. However, Clostridium perfringens strains often carry multiple conjugative toxin or antibiotic resistance plasmids that are closely related and encode similar Rep proteins. In many bacteria, plasmid partitioning upon cell division involves a ParMRC system; in C. perfringens plasmids, there are approximately 10 different ParMRC families, with significant differences in amino acid sequences between each ParM family (15% to 54% identity). Since plasmids carrying genes belonging to the same ParMRC family are not observed in the same strain, these families appear to represent the basis for plasmid compatibility in C. perfringens. To understand this process, we examined the key recognition steps between ParR DNA-binding proteins and their *parC* binding sites. The ParR proteins bound to sequences within a *parC* site from the same ParMRC family but could not interact with a *parC* site from a different ParMRC family. These data provide evidence that compatibility of the conjugative toxin plasmids of C. perfringens is mediated by their *parMRC*-like partitioning systems. This process provides a selective advantage by enabling the host bacterium to maintain separate plasmids that encode toxins that are specific for different host targets.

## INTRODUCTION

Low-copy-number plasmids usually require an active partitioning system to ensure that they are faithfully inherited by daughter cells upon cell division (1). Type II (ParMRC) plasmid partitioning systems include three components: *parC*, a plasmid-borne centromere; ParM, an actin-like ATPase that forms filaments in the presence of ATP or GTP; and ParR, a DNA-binding adaptor protein that binds to *parC* (2–6). ParMRC systems stabilize the inheritance of plasmids by positioning them on either side of the cell septum prior to cell division.

ParR proteins are typically ribbon-helix-helix proteins that bind to direct repeats within *parC*, either as a dimer or as a dimer of dimers (5, 7–10). The *parC* centromere usually consists of a series of direct repeats upstream of the *parM* gene; however, its precise genetic structure differs between plasmids. Binding of ParR acts to seed the formation of a higher-order solenoid-shaped structure, termed the segrosome, where the DNA wraps around ParR, leaving a core of ParM interaction sites (9, 10). Polymerizing ParM filaments then link the ParR-*parC* complexes of two sister plasmids and push them to either cell pole (2, 3, 11–13). The initial step, in which ParR recognizes and interacts with *parC*, is important in determining partition specificity between plasmids.

Plasmid incompatibility generally occurs when two coresident plasmids encode the same essential replication or partitioning machinery (14). Most studies to date have focused on the partition specificity and incompatibility mediated by type I (ParABS) partitioning systems (15–19); there is only limited evidence that partition-mediated incompatibility can also be facilitated by ParMRC-like partitioning systems (19, 20).

**FIG 1:**
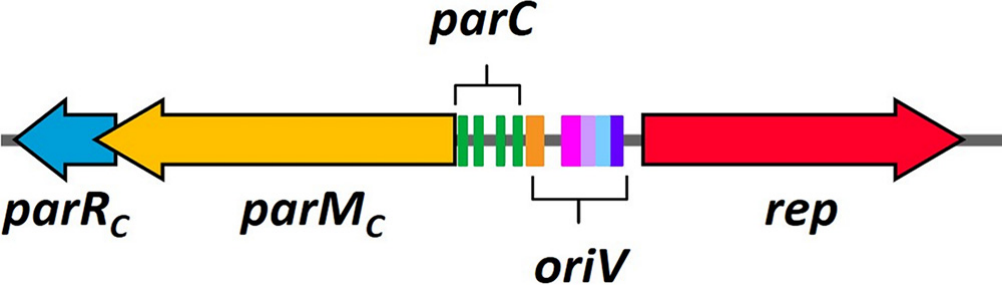
Replication and *parMRC* locus of pCW3. *parM_C_* is shown in yellow, *parR_C_* is shown in blue, the *parC_C_* site (four direct repeats shown in green) is upstream of *parM_C_*, and the five inverted repeats (IR) of *oriV* (IR1 in orange, IR2 in pink, IR3 in lavender, IR4 in blue, and IR5 in purple) are shown upstream of *rep*, which is indicated by the red arrow.

In this study, we focused on partition-mediated incompatibility in Clostridium perfringens, a Gram-positive pathogen. In humans and animals, C. perfringens produces an extensive range of toxins, which it uses to cause diseases that range from mild food poisoning to often fatal infections such as clostridial myonecrosis, enteritis, and enterotoxemia (21). Most C. perfringens toxins are encoded on large, low-copy-number, conjugative plasmids (22) that are similar to the tetracycline resistance plasmid pCW3 (22–29). These plasmids have approximately 35 kb of sequence similarity that includes the *tcp* conjugation locus and genes involved in replication, regulation, and stable plasmid maintenance (Fig. 1) (23, 24, 28, 30–33). Even though these plasmids have similar replication regions, including a highly conserved replication protein, C. perfringens strains frequently carry up to five discrete plasmids (24, 34). This phenomenon is typified by the avian necrotic enteritis isolate EHE-NE18, which stably maintains three large, closely related conjugative plasmids with Rep proteins that have 98% amino acid sequence identity (24, 34).

Bioinformatic analysis has revealed the presence of at least 10 families of ParMRC partitioning systems (ParMRC_A_ to ParMRC_J_) in these pCW3-like plasmids. The ParM components have >90% amino acid sequence identity within a family and 15 to 54% amino acid sequence identity between families, and the ParR and *parC* components show a similar trend (34). A representative of the ParMRC_B_ family was shown to be a true partitioning system, as addition of this partitioning system to an unstable minireplicon was sufficient to stabilize its inheritance in Escherichia coli (35). Strains of C. perfringens do not usually carry plasmids that encode the same ParMRC partitioning system (24, 28, 34), which suggests that these plasmids have evolved different partition specificities to ensure that they are stably maintained within a single C. perfringens cell.

We have shown that pCW3-like plasmids with identical partitioning systems cannot be maintained in a single cell without selection, whereas plasmids with ParMRC systems from different families are stably maintained in C. perfringens cells (36). This finding suggested that differences in ParMRC plasmid partitioning systems were responsible for determining plasmid incompatibility between similar replicons and dictated which plasmid combinations could coexist in an isolate. In this study, we have utilized surface plasmon resonance (SPR) and analytical ultracentrifugation (AUC) to demonstrate that differences in the ParR and *parC* components of these partitioning families are reflected in their binding specificity, providing the essential biochemical evidence for the critical role of the ParMRC system in determining plasmid compatibility in C. perfringens.

## RESULTS

### Identification of the pCW3 ParR_C_ binding site.

The recognition steps between ParM, ParR, and *parC* components both within and between different families of *parMRC* systems are likely to be key drivers in determining the specificity of the partition reaction and therefore plasmid incompatibility in C. perfringens. The ParR-*parC* interaction is of particular interest because this is the first recognition step in the partitioning reaction (8, 10, 11) and is responsible for the incompatibility phenotype in some other plasmids (20).

**FIG 2:**
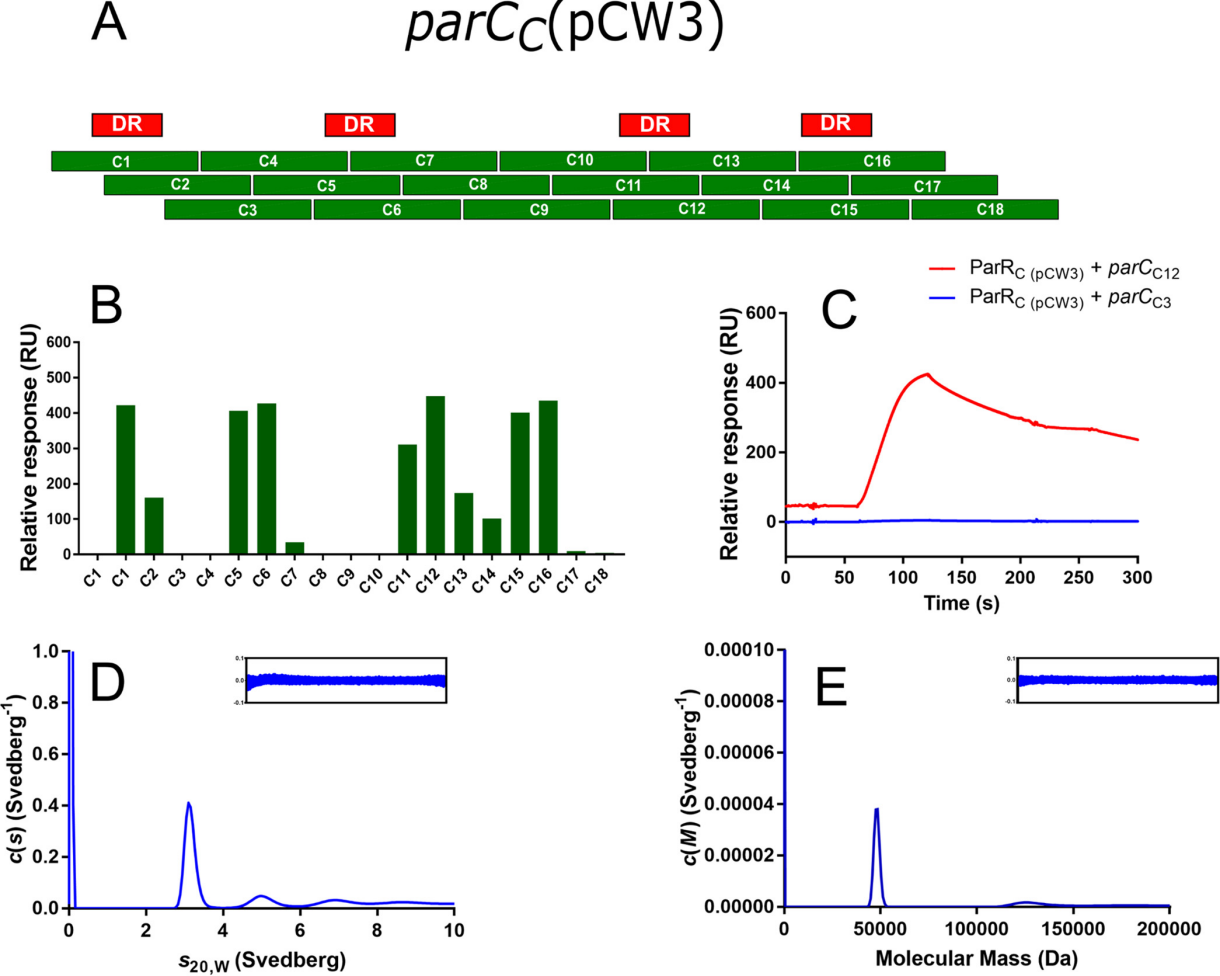
ParR_C_(pCW3) binds to a cognate *parC_C_*(pCW3) sequence. (A) Schematic of the *parC_C_*(pCW3) fragment array, which consists of 30-bp fragments that overlap by 20 bp; direct repeats are indicated above the fragment array in red. (B) Representative ParR_C_(pCW3) binding to the *parC_C_*(pCW3) fragment array as determined by SPR. The first instance of C1 on the graph indicates a no-ParR control. Binding stability measurements were recorded 10 s after the end of sample injection. (C) Representative SPR binding curves for ParR_C_(pCW3) and *parC_C_*(pCW3) fragments. ParR_C_(pCW3) plus the C3 binding curve is shown in blue, and ParR_C_(pCW3) plus the C12 binding curve is shown in red. AUC sedimentation velocity experiments were also conducted on ParR_C_(pCW3), *parC_C_*(pCW3) fragment C5, and ParR_C_(pCW3) and *parC_C_*(pCW3) fragment C5 in combination. (D) Continuous sedimentation coefficient distribution [*c*(*s*)] as a function of normalized sedimentation coefficient (*s*_20,W_) for ParR_C_(pCW3). (E) Continuous mass distribution *c*(*M*) distribution as a function of molecular mass for ParR_C_(pCW3).

SPR was employed to interrogate ParR-*parC* interactions. We first chose to examine the interaction between ParR_C_ and *parC_C_* from pCW3, as pCW3 is the best-characterized conjugative antimicrobial resistance plasmid in C. perfringens (30). To perform SPR, a recombinant His_6_-tagged ParR_C_(pCW3) protein was expressed in E. coli and purified (see Fig. S1 in the supplemental material). A series of overlapping oligonucleotide fragments were designed (37) based on the 192-bp *parC_C_* region of pCW3 (Fig. 1). These oligonucleotides were annealed to produce a fragment array consisting of 18 double-stranded *parC_C_* fragments (designated C1 to C18) (Fig. 2A). The stability and specificity of the ParR-*parC* interaction were assessed by challenging each *parC_C_* fragment with ParR_C_(pCW3) (Fig. 2B and C). Strong interactions (a binding stability value of >100 response units [RU]) between ParR_C_(pCW3) and fragments C1 (256 RU), C5 (249 RU), C6 (282 RU), C11 (154 RU), C12 (348 RU), C15 (217 RU), and C16 (311 RU) were observed. Weaker interactions (a stability value between baseline and 100 RU) were also noted for fragments C2 (54 RU), C7 (9 RU), C13 (48 RU), and C14 (42 RU). The strong interactions that were observed between the *parC_C_*(pCW3) fragments and ParR_C_ were mapped to the *parC_C_*(pCW3) nucleotide sequence, which showed that binding corresponded with the presence of four conserved 17-bp direct repeats (5′-AAACATCACAATTTTAC).

10.1128/mbio.01356-22.1 FIG S1 Purified recombinant ParR proteins. ParR proteins were purified and analyzed using a 15% polyacrylamide gel stained with Coomassie brilliant blue. 1, Bio-Rad protein standards; 2, ParR_B_(pJIR4165); 3, ParR_B_(pJGS1987B); 4, ParR_C_(pCW3); 5, ParR_C_(pJGS1987C); 6, ParR_D_(pJGS1987D); 7, ParR_D_(pJIR3118). Download FIG S1, DOCX file, 0.6 MB. Copyright © 2022 Watts et al. 2022 Watts et al. https://creativecommons.org/licenses/by/4.0/ This content is distributed under the terms of the Creative Commons Attribution 4.0 International license.

**FIG 3:**
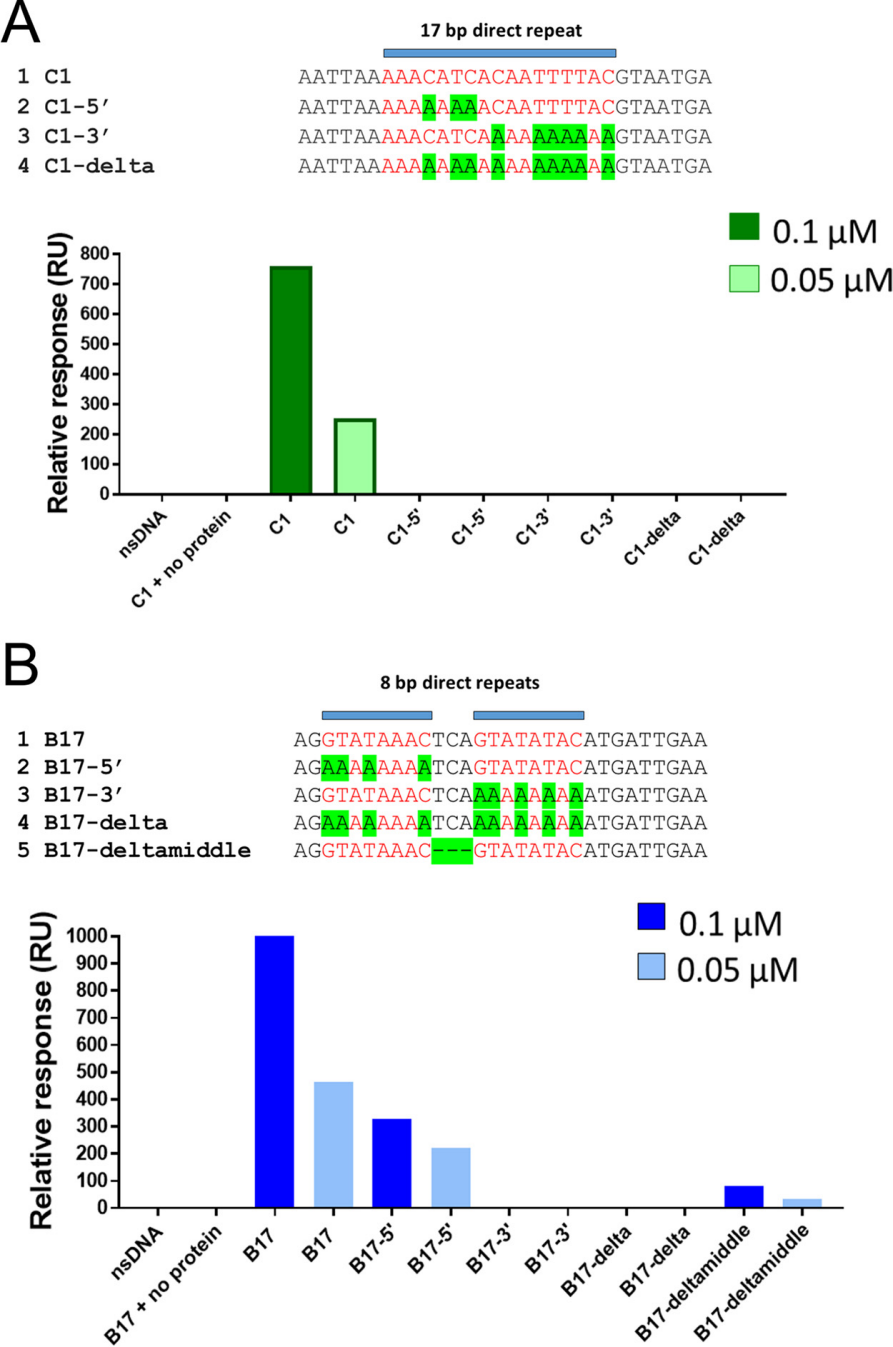
ParR_C_(pCW3) and ParR_B_(pJIR4165) bind to direct repeats within cognate *parC* sites. (A) Items 1 to 4 show an alignment of the mutant *parC_C_* fragments (C1-5′, C1-3′, and C1-delta) compared to *parC_C_* (C1). SPR data of ParR_C_(pCW3) interaction with *parC_C_* mutant fragments are shown in the graph in green (0.1 μM) and light green (0.05 μM). (B) Items 1 to 5 show an alignment of the mutant *parC_B_* fragments (B17-5′, B17-3′, B17-delta, and B17-deltamiddle) compared to *parC_B_* (B17). SPR data of ParR_B_(pJIR4165) interaction with *parC_B_* mutant fragments are shown in the graph in blue (0.1 μM) and light blue (0.05 μM). All binding stability measurements were recorded 10 s after the end of sample injection.

Subsequently, a series of mutated *parC_C_*(pCW3) (C1) fragments were constructed to assess the importance of the 17-bp repeat to ParR_C_(pCW3) binding. Three altered fragments were constructed in which the cytosine and thymine bases in the 17-bp repeat were replaced with adenine (Fig. 3A), resulting in the generation of the fragments C1-5′, C1-3′, and C1-delta, which had four nucleotide changes, five nucleotide changes, and nine nucleotide changes in the 17-bp repeat, respectively. Analysis of the interaction between ParR_C_(pCW3) and these fragments revealed that any of these changes to the 17-bp repeat led to loss of ParR binding (Fig. 3A). The SPR results also indicated that a single fragment with the conserved *parC_C_* repeat was sufficient for ParR_C_ binding.

AUC sedimentation velocity experiments were used to obtain insight into the multimeric state of ParR_C_ in solution. The interaction between ParR_C_(pCW3) and the *parC_C_*(pCW3) fragment C5 was chosen for interrogation, as the C5 fragment had a centrally located direct repeat and showed strong binding to ParR_C_(pCW3) by SPR. The results showed that ParR_C_(pCW3) primarily sedimented as a single species, with a sedimentation coefficient (*s*_20,W_) of 3.1 S (Fig. 2D), which corresponded to a molecular mass of 48 kDa (Fig. 2E). The molecular mass of His_6_-tagged ParR_C_(pCW3) as predicted from the amino acid sequence is 10.9 kDa, suggesting that ParR_C_(pCW3) exists as a tetramer in solution. The *parC_C_*(pCW3) C5 fragment sedimented as a single species, with a sedimentation coefficient of 2.7 S (Fig. S2A). When ParR_C_(pCW3) and *parC_C_*(pCW3) C5 were combined prior to centrifugation, a distinct shift in sedimentation coefficient to 4.2 S was observed (Fig. S2A), which was consistent with binding in a 1:1 ratio of ParR_C_(pCW3) complex (four molecules) to each *parC_C_*(pCW3) binding site. Note that samples that are subjected to AUC are detected using UV light. However, ParR_C_ does not contain any tryptophan residues and therefore fluoresces poorly when exposed to UV light. To compensate for this limitation, AUC was conducted using much higher concentrations of ParR_C_ than were used in SPR. At these concentrations (>25 μM), ParR_C_ also interacted with a nonspecific DNA control [*parC_C_*(pCW3) (C9)] (Fig. S2A). These results confirmed that ParR_C_(pCW3) and *parC_C_*(pCW3) (C5) could interact in solution, which was consistent with the results obtained via SPR but showed that ParR_C_(pCW3) can also interact nonspecifically at high concentrations. In addition, the stoichiometry of binding at high concentration may not reflect physiologically relevant complexes; we therefore drew upon our SPR binding data to determine the stoichiometry of binding between 0.1 μM ParRC and a *parC* fragment containing the predicted binding site (Table S6). SPR showed that the association between ParR_C_(pCW3) and bound *parC_C_*(pCW3) (C1, C5, C6, C11, C12, C15, and C16) was approximately 2:1 (ParR to *parC*) except for the fragments C11 and C15, which do not have nucleotides immediately downstream of the direct repeat. This result suggests that interaction between a ParR dimer and its cognate binding site occurs when downstream context is provided.

10.1128/mbio.01356-22.2 FIG S2 Binding of ParR_C_(pCW3) with *parC_C_*(pCW3) fragments. (A) Analytical ultracentrifugation shows that at high concentrations, ParR_C_(pCW3) interacts nonspecifically with *parC_C_*(pCW3) fragments. Analytical ultracentrifugation of ParR_C_(pCW3) mixed with *parC_C_*(pCW3) fragments either containing the direct repeat binding site (C5 shown in red) or without the direct-repeat binding site (C9 shown in blue) was performed. The first portion shows *parC_C_*(pCW3) fragments alone, the middle portion shows ParR_C_(pCW3) plus *parC_C_*(pCW3) in a 2:1 ratio, and the final portion shows ParR_C_(pCW3) plus *parC_C_*(pCW3). The shift in *s*_20_,_W_ shows that ParR interacts with both *parC* fragments, indicating that ParR_C_ binds nonspecifically at high concentrations. (B) Electrophoretic mobility shift assay shows that ParRC binds specifically to *parC*_C_ fragments containing 17-bp direct repeats. F, free DNA; C, ParR-*parC_C_* (C5) complex; C5 unlabelled, unlabeled *parC_C_* (C9) in excess (200×) nonspecific competitor; unlabelled C5, unlabeled *parC_C_* (C5) in excess (200×) specific competitor. 1, no-protein control; 2, labeled *parC_C_* (C5) fragment plus ParR_C_(pCW3) (ratio of 1 pmol of *parC_C_* [C5] to 1 pmol of protein); 3, labeled *parC_C_* (C5) fragment plus ParR_C_(pCW3) (ratio of 1 pmol of *parC_C_* [C5] to 4 pmol of protein); 4, labeled *parC_C_* (C5) plus unlabeled C9; 5, labeled *parC_C_* (C5) plus NSC (C9) plus ParR_C_(pCW3) (ratio of 1 pmol of *parC_C_* [C5] to 1 pmol of protein); 6, labeled *parC_C_* (C5) plus NSC (C9) plus ParR_C_(pCW3) (ratio of 1 pmol of *parC_C_* [C5] to 4 pmol of protein); 7, no protein; 8, labeled *parC_C_* (C5) fragment plus ParR_C_(pCW3) (ratio of 1 pmol of *parC_C_* [C5] to 1 pmol of protein); 9, ParR_C_(pCW3) plus labeled *parC_C_* (C5) plus unlabeled C5. Download FIG S2, DOCX file, 0.6 MB. Copyright © 2022 Watts et al. 2022 Watts et al. https://creativecommons.org/licenses/by/4.0/ This content is distributed under the terms of the Creative Commons Attribution 4.0 International license.

10.1128/mbio.01356-22.10 TABLE S6 ParR_C_-*parC_C_* (pCW3) predicted binding site stoichiometry as detected via SPR. Download Table S6, DOCX file, 0.01 MB. Copyright © 2022 Watts et al. 2022 Watts et al. https://creativecommons.org/licenses/by/4.0/ This content is distributed under the terms of the Creative Commons Attribution 4.0 International license.

To reconcile the differences in binding specificities observed between the SPR results and the AUC data, we performed electrophoretic mobility shift assays (EMSA) with ParR_C_(pCW3), *parC_C_*(pCW3) (C5), and *parC_C_*(pCW3) (C9). A specific shift was observed when ParR_C_ was mixed with labeled C5 DNA at a ratio of 1 pmol to 1 pmol or 1 pmol to 4 pmol, compared to a no-protein control, which showed no shift (Fig. S2B). Similarly, when the unlabeled nonspecific inhibitor DNA *parC_C_*(pCW3) (C9) was included in the reaction in excess (200×), a specific shift between ParR_C_(pCW3) and the labeled *parC_C_*(pCW3) (C5) DNA was observed (Fig. S2B). In contrast, upon the addition of unlabeled specific competitor *parC_C_*(pCW3) (C5) DNA, the specific shift between ParR_C_(pCW3) and labeled *parC_C_*(pCW3) (C5) was disrupted (Fig. S2B).

### ParR homologues cannot bind to noncognate *parC* centromeres from a different phylogenetic ParMRC family.

**FIG 4:**
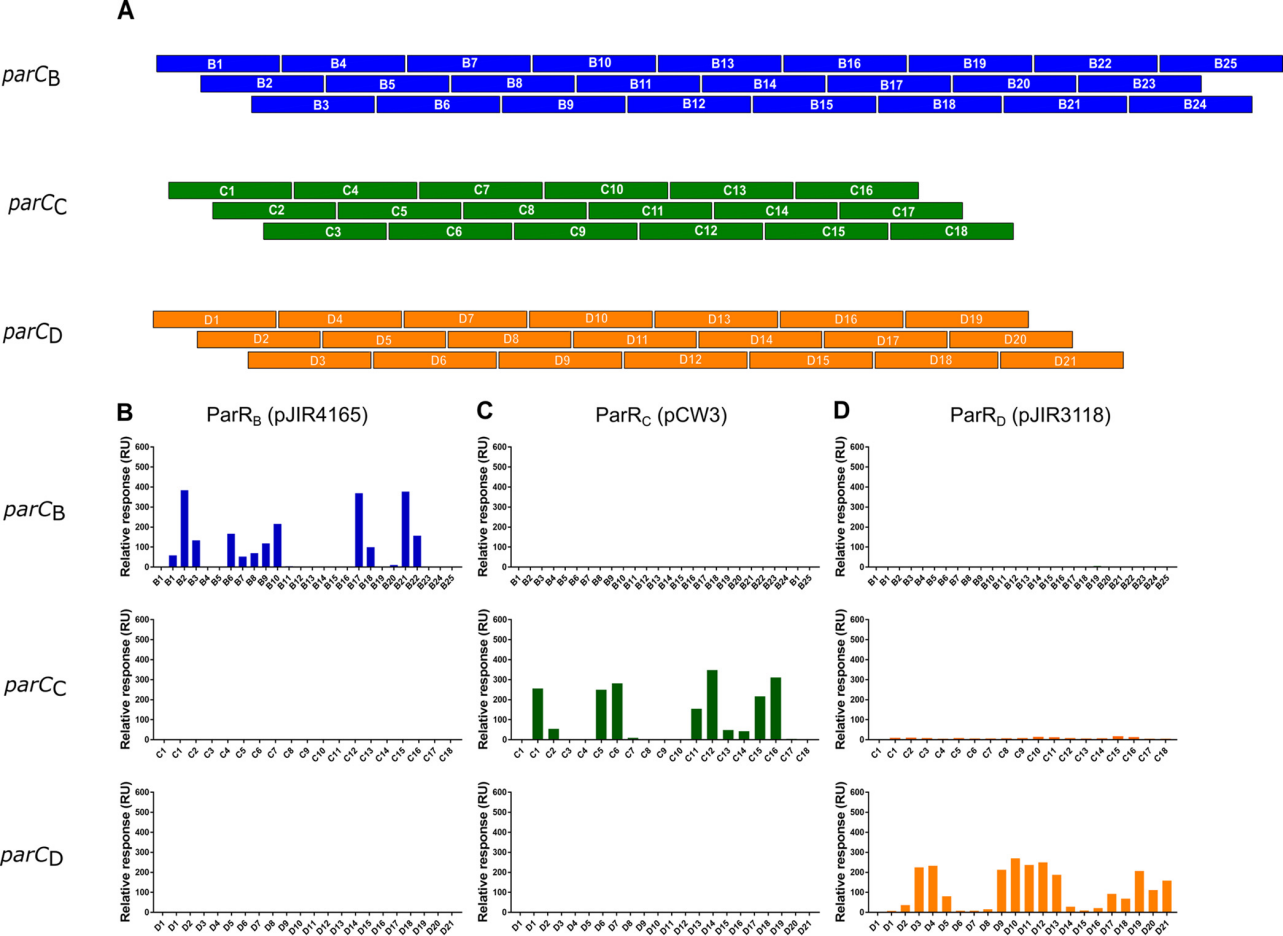
SPR analysis demonstrated that ParR homologues bind to their cognate *parC* sites. (A) Schematic of *parC* overlapping fragments. *parC_B_*(pJIR4165), *parC_C_*(pCW3), and *parC_D_*(pJIR3118) fragment arrays were constructed to test binding of ParR homologues to each *parC* region. All fragment arrays consisted of 30-bp oligonucleotides with 20 bp of overlapping sequence and were designed using POOP. Antisense oligonucleotides were constructed with the ReDCaT linker sequence present at the 3′ end of each fragment in the diagram above. Oligonucleotides were annealed before being captured onto the ReDCaT-primed streptavidin (SA) chip via the complementary base-pairing between the ReDCaT linker and the complementary ReDCaT sequence on the Biacore T200 chip. (B) SPR profiles obtained when ParR_B_(pJIR4165) was tested against *parC_B_*(pJIR4165) (blue), *parC_C_*(pCW3) (green), and *parC_D_*(pJIR3118) (orange). (C) ParR_C_(pCW3) binding profiles. (D) ParR_D_(pJIR3118) binding profiles. The first lane in every binding graph shows a no-protein control with the fragments C1, B1, and D1. All binding stability measurements were recorded 10 s after the end of sample injection.

To determine if the interaction of ParR and *parC* components is ParMRC family specific, two more ParR and *parC* families were included in the SPR analysis. ParR_B_ from pJIR4165 and ParR_D_ from pJIR3118 have 11% and 26% amino acid sequence identity to ParR_C_(pCW3), respectively, and were expressed and purified (Fig. S1). In addition, *parC_B_*(pJIR4165) and *parC_D_*(pJIR3118) fragment arrays were synthesized to yield fragments B1 to B25 and D1 to D21 (Fig. 4A); these regions, respectively, have 45% and 47% nucleotide sequence identity to *parC_C_*(pCW3) (Table S1). ParR_B_(pJIR4165), ParR_C_(pCW3), and ParR_D_(pJIR3118) were tested against each *parC* fragment array [*parC_B_*(pJIR4165), *parC_C_*(pCW3), and *parC_D_*(pJIR3118)] in separate SPR experiments (Fig. 4).

10.1128/mbio.01356-22.5 TABLE S1 Amino acid sequence identity matrix of ParR homologues and nucleotide sequence identity matrix of *parC* regions. Download Table S1, DOCX file, 0.01 MB. Copyright © 2022 Watts et al. 2022 Watts et al. https://creativecommons.org/licenses/by/4.0/ This content is distributed under the terms of the Creative Commons Attribution 4.0 International license.

The results showed that each ParR homologue bound only to its cognate *parC* fragment array. ParR_B_(pJIR4165) bound to 12 *parC_B_*(pJIR4165) fragments, with the strongest binding (binding stability value of >300 RU) to fragments B2 (383 RU), B17 (368 RU), and B21 (377 RU) (Fig. 4B). The *parC_C_*(pCW3) site had a clear correlation between binding and the direct-repeat structures, but the *parC_B_*(pJIR4165) region was more complex.

**FIG 5:**
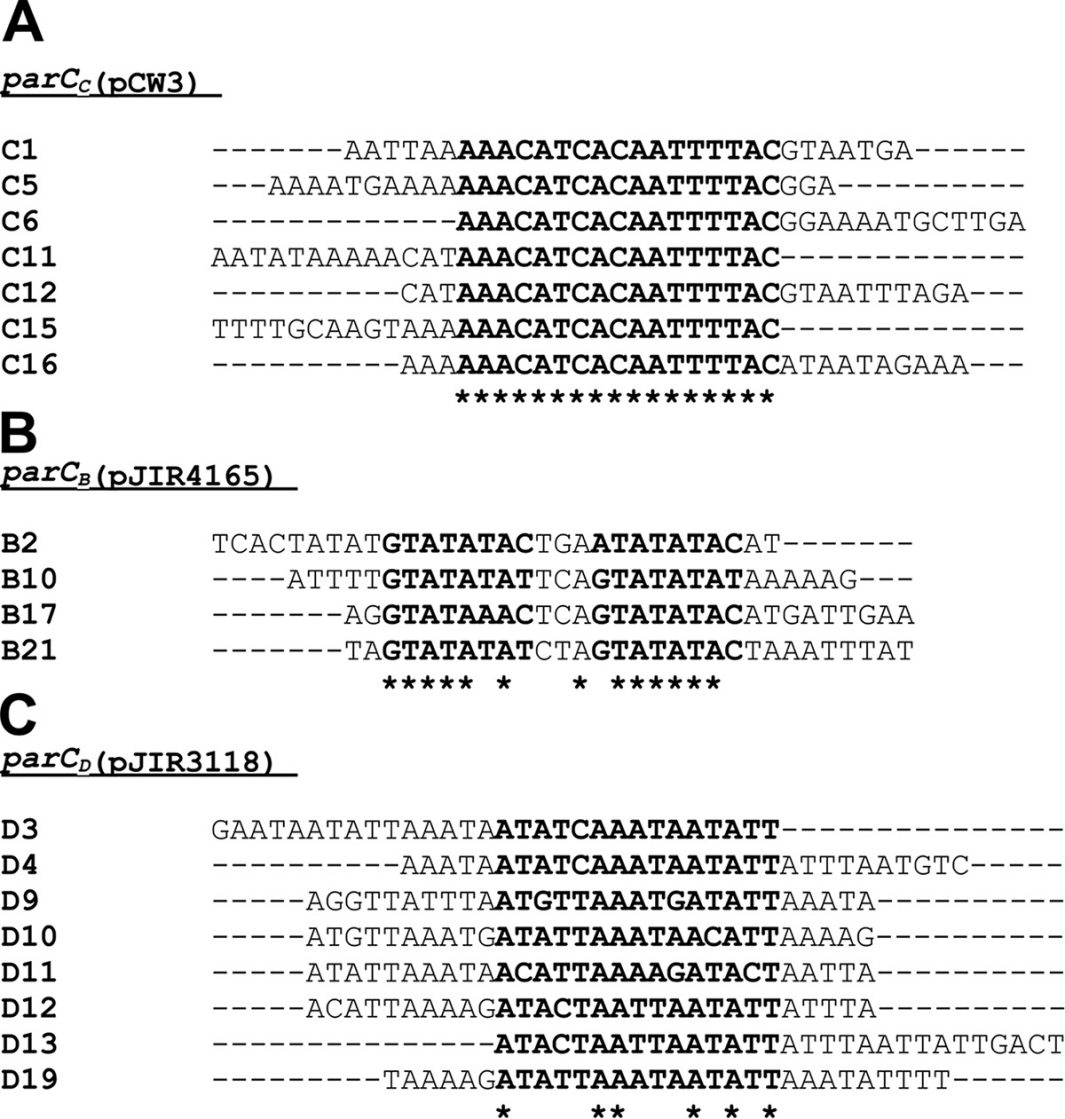
Sequence alignment of *parC* fragment that interact with ParR. The *parC* fragments that each ParR homologue interacted with were aligned using ClustalΩ to identify conserved binding sites; the predicted binding site is shown in bold, and identical residues are indicated by the asterisks. (A) *parC_C_*(pCW3) fragment alignment; (B) *parC_B_*(pJIR4165) fragment alignment; (C) *parC_D_*(pJIR3118) fragment alignment.

The *parC_B_*(pJIR4165) site consists of several different direct repeats and two inverted-repeat structures, and many of these structures overlap. Therefore, mapping of ParR_B_(pJIR4165) binding to the *parC_B_*(pJIR4165) region did not indicate a clear ParR_B_(pJIR4165) binding site. The fragments that displayed the highest SPR response were aligned using Clustal Omega to identify conserved sequences that were required for ParR_B_(pJIR4165) binding (Fig. 5B). Two imperfect 8-bp direct repeats that were separated by 3 bp were identified in each fragment (Fig. 5B). Several mutated *parC_B_*(pJIR4165) (B17) fragments then were constructed to assess the importance of these 8-bp repeats, and the spacing between them, to ParR_B_(pJIR4165) binding (Fig. 3B). Fragments were constructed that had the guanine, cytosine, and thymine bases in either the 5′ or the 3′ 8-bp repeat replaced with adenine, and in another fragment, the 3-bp spacing between the repeats was deleted (Fig. 3B). Analysis of ParR_B_ (pJIR4165) binding to these fragments revealed that these changes to the B17-3′ 8-bp repeat (GTATAATC) resulted in a loss of binding. In contrast, replacement of the B17-5′ repeat with adenines resulted in a reduced level of ParR_B_ binding. Finally, removal of the 3 bp between the two repeats showed a response comparable to that for the B17-3′ replacement fragment, i.e., loss of binding. This result demonstrated that this spacing region is important for the recognition and binding of ParR_B_ to *parC_B_* (Fig. 3B).

ParR_C_(pCW3) was tested against the *parC_B_*(pJIR4165) and *parC_D_*(pJIR3118) fragment arrays and showed no interaction with these noncognate sequences (Fig. 4C). SPR analysis of the *parC_D_*(pJIR3118) fragment array with its cognate ParR_D_(pJIR3118) protein showed strong binding stability values (>100 RU) with fragments D3 (225 RU), D4 (232 RU), D9 (213 RU), D10 (270 RU), D11 (236 RU), D12 (250 RU), and D13 (187 RU) and weaker interactions (below 100 RU) with eight other oligonucleotide fragments (Fig. 4D). Inspection of the *parC_D_*(pJIR3118) region revealed several different AT-rich direct- and inverted-repeat structures.

**FIG 6:**
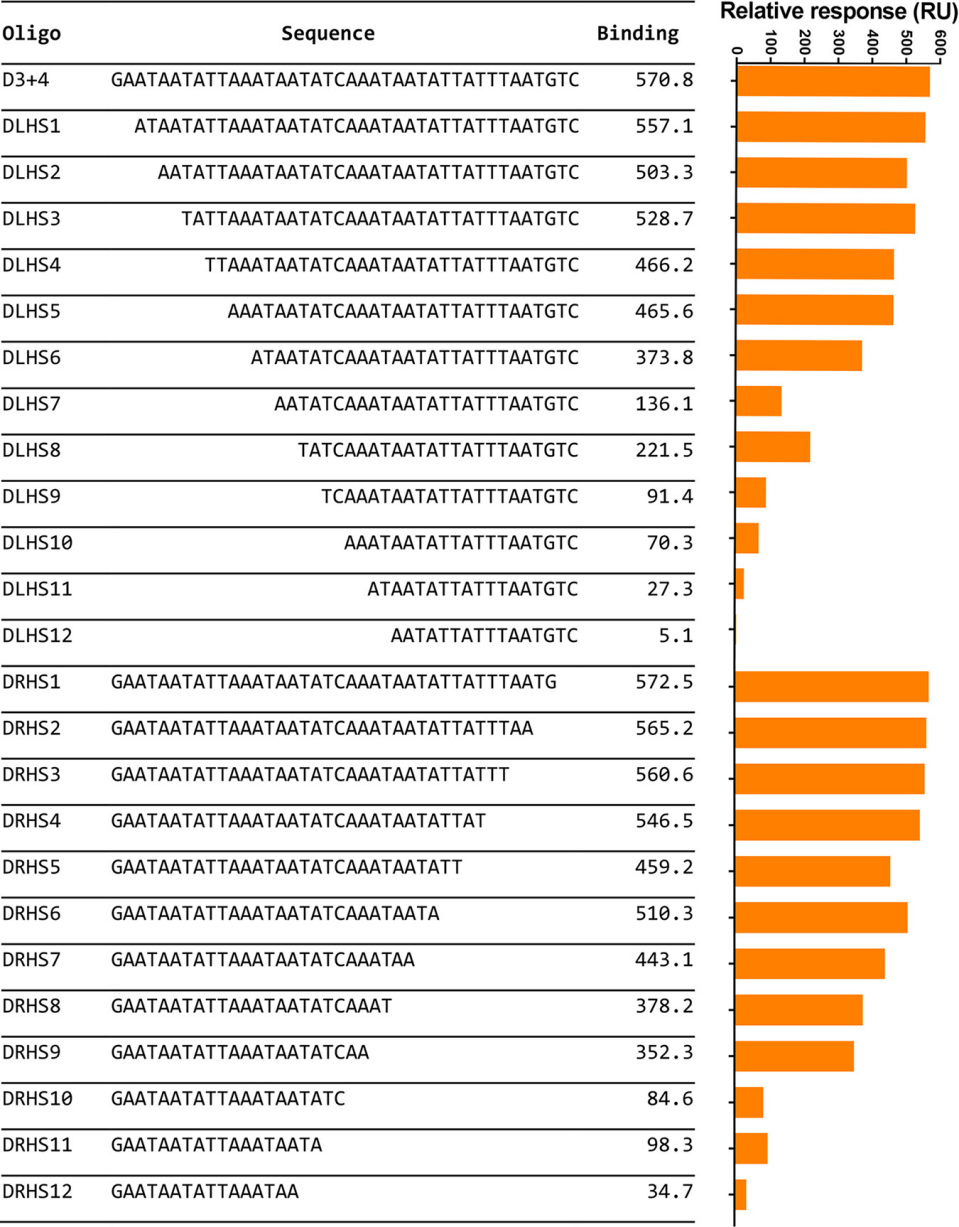
*parC_D_*(pJIR3118) SPR footprinting reveals ParR_D_(pJIR3118) binding site. Fragments with 2-bp deletions of the fragment D3 plus 4 from either right-hand side or 5′ (RHS) or left-hand side or 3′ (LHS) were constructed and tested for the ability to bind ParR_D_(pJIR3118). Oligonucleotide name and nucleotide sequence are indicated in the table. Relative binding response is indicated for each fragment by the values in column three and on the graph in orange. All binding stability measurements were recorded 10 s after the end of sample injection.

In contrast to the other ParR proteins, ParR_D_ proteins have a high pI value (~9 compared to ~4 to 5), and therefore, these proteins may bind more promiscuously to DNA than other ParR proteins. These nonspecific interactions were minimized by adding the blocking agent dextran to the SPR sample buffer; however, mapping of the ParR_D_(pJIR3118) interactions did not give a clear indication of the specific ParR_D_ binding site. To further resolve the ParR_D_ binding site, an SPR footprinting approach was used. A fragment that was composed of *parC_D_* fragments D3 and D4 and a series of sequential deletion derivatives based on this fragment with 2-bp deletions from either the 5′ or 3′ end were constructed (D0, DLHS1 to -12, and DRHS1 to -12) (Fig. 6). The ability of ParR_D_ to bind to these fragments was tested, and the results showed that ParR_D_ binding was greatly reduced when the AT-rich sequence 5′-ATAATATCAA was disrupted, indicating that this sequence is important for binding. Mapping this sequence to other strong binding fragments resulted in the identification of a partially conserved AT-rich ParR_D_ binding sequence (Fig. 5C).

ParR_D_(pJIR3118) did not interact with the *parC_B_*(pJIR4165) fragment array and showed only very weak interactions with most of the fragments from the *parC_C_*(pCW3) array (stability values between 5 and 15 RU above baseline). These interactions are likely to be nonspecific, as a low level of binding was observed for all fragments, including the reusable DNA capture technique (ReDCaT) control fragment. The nonspecific interactions were minimized by the addition of dextran to the SPR sample buffer, which had no effect on binding to the *parC_D_*(pJIR3118) fragments. Overall, these results highlight the specificity of the ParR-*parC* interactions, where ParR homologues only bind to their cognate *parC* component and have either no interaction or very weak interfamily interactions.

### ParR homologues recognize and bind noncognate *parC* fragment arrays from the same ParMRC family.

Our earlier work suggested that ParMRC components from the same family would be able to interact with one another, thus leading to interference with the partition process and plasmid incompatibility (36). To provide biochemical evidence for this hypothesis, three different ParR homologues (ParR_B_, ParR_C_, and ParR_D_) from the C. perfringens strain JGS1987 were expressed, purified (Fig. S1), and used to assess their capacity to facilitate intrafamily interactions. There is an unpublished whole-genome shotgun sequence available for strain JGS1987 (GenBank accession number: ABDW00000000), and it was chosen for analysis because an earlier bioinformatic survey revealed that this strain was particularly rich in *parMRC* genes (34). The JGS1987 sequence contains seven different *parM* alleles, which suggests that there may be seven potential plasmids present in this strain. Since these plasmid sequences had not been closed or given plasmid names, each putative plasmid was designated based on the strain of origin and the *parMRC* genes associated with that contig, yielding pJGS1987B, pJGS1987C, pJGS1987D, etc. The JGS1987 ParR_B_, ParR_C_, and ParR_D_ homologues have 96%, 96%, and 95% amino acid identity to the equivalent ParR_B_(pJIR4165), ParR_C_(pCW3), and ParR_D_(pJIR3118) proteins (Table S1) (38). The corresponding JGS1987 *parC* regions also show high levels (82% to 91%) of nucleotide sequence identity to their equivalent homologues (Table S1 and Fig. S3). We postulated that the respective JGS1987-derived ParR proteins would cross-react with *parC* arrays from other members of the same ParMRC family. To examine this hypothesis, we tested the existing suite of *parC* fragment arrays with the purified ParR homologues from JGS1987.

10.1128/mbio.01356-22.3 FIG S3 Conserved repeats in *parC* regions. Intrafamily alignments of each *parC* region. (A) Nucleotide sequence alignment between *parC_B_*(pJIR4165) and *parC_B_*(pJGS1987B); putative binding sites direct repeats are shown in green. (B) Nucleotide sequence alignment between *parC_C_*(pCW3) and *parC_C_*(pJGS1987C); conserved direct repeats are shown in pink. (C) Nucleotide sequence alignment of *parC_D_*(pJIR3118) and *parC_D_*(pJGS1987D); direct repeats are shown in blue. Download FIG S3, DOCX file, 0.1 MB. Copyright © 2022 Watts et al. 2022 Watts et al. https://creativecommons.org/licenses/by/4.0/ This content is distributed under the terms of the Creative Commons Attribution 4.0 International license.

**FIG 7:**
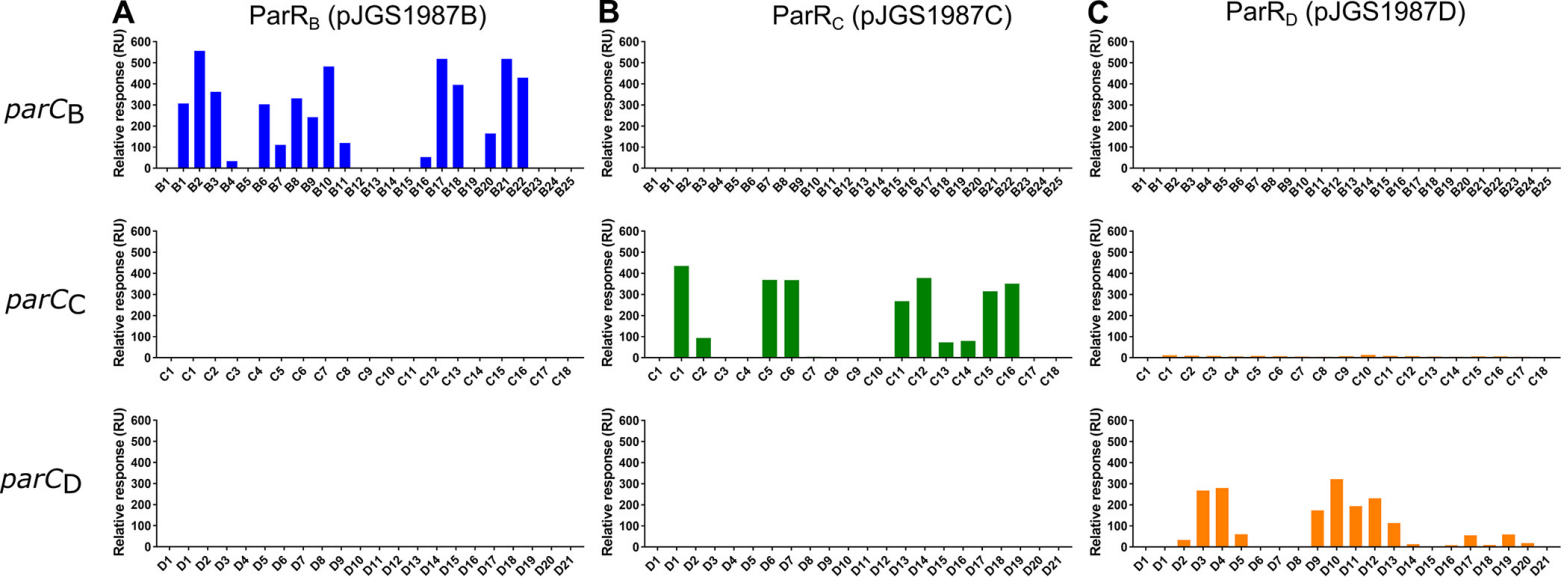
JGS1987 ParR homologues bind to noncognate *parC* from the same family. ParR_B_, ParR_C_, and ParR_D_ homologues from C. perfringens isolate JGS1987 were tested against *parC_B_*(pJIR4165), *parC_C_*(pCW3), and *parC_D_*(pJIR3118) fragment arrays, and binding stability was measured using SPR. (A) ParR_B_(pJGS1987B) binding profiles when used to challenge *parC_B_*(pJIR4165) (blue), *parC_C_*(pCW3), and *parC_D_*(pJIR3118). (B) ParR_C_(pJGS1987C) binding profiles (blue). (C) ParR_D_(pJGS1987D) binding profiles (orange). The first fragment in every graph shows a no-protein control. All binding stability measurements were recorded 10 s after the end of sample injection.

The results showed that the JGS1987 ParR homologues interacted with noncognate *parC* fragment arrays from the same ParMRC family but not with noncognate *parC* fragments from different families (Fig. 7). ParR_B_(pJGS1987B) interacted with *parC_B_*(pJIR4165) with a binding pattern comparable to that of ParR_B_(pJIR4165) (Fig. 7A). Strong binding stability (>200 RU) scores were recorded for interactions between ParR_B_(pJGS1987B) and *parC_B_*(pJIR4165) fragments B1, B2, B3, B6, B8, B9, B10, B17, B18, B20, B21, B22, and B25. Weaker binding stability scores were seen for fragments B4, B7, B11, B16, and B23.

Similarly, ParR_C_(pJGS1987C) interacted only with *parC_C_*(pCW3), with the same binding pattern as observed for ParR_C_(pCW3) (Fig. 7B). High binding stability (>200 RU) scores were recorded for interactions between ParR_C_(pJGS1987C) and *parC_C_*(pCW3) fragments C1, C5, C6, C11, C12, and C15. Weaker binding stability scores were recorded for C2, C13, and C14.

ParR_D_(pJGS1987D) only interacted with its noncognate, but intrafamily, array from *parC_D_*(pJIR3118) (Fig. 7C). Strong binding stability scores were recorded for interactions between ParR_D_(pJGS1987D) and *parC_D_*(pJIR3118) fragments D3, D4, D9, D10, D11, D12, D13, and D19, whereas weaker binding stability scores were recorded for fragments D2, D5, D14, D16, D17, D18, and D20. Representative binding curves for each ParR-*parC* interaction pair are presented in Fig. S4. These data showed that ParR homologues interacted with noncognate *parC* fragments from the same phylogenetic ParMRC family, thus confirming a subset of the bioinformatically derived phylogenetic groups of these homologues.

10.1128/mbio.01356-22.4 FIG S4 Representative SPR binding curves for each ParR homologue and cognate *parC* fragment interactions. Representative binding curves for each ParR-*parC* pair are shown. Panel A shows representative binding curves for ParR_B_ homologues, panel B shows representative binding curves for ParR_C_ homologues, and panel C shows representative binding curves for ParR_D_ homologues. The red curves represent a binding interaction; blue curves represent a nonbinding interaction. The origins of the ParR and *parC* fragments are indicated above each graph. Download FIG S4, DOCX file, 0.6 MB. Copyright © 2022 Watts et al. 2022 Watts et al. https://creativecommons.org/licenses/by/4.0/ This content is distributed under the terms of the Creative Commons Attribution 4.0 International license.

## DISCUSSION

In this study, we have demonstrated that ParR homologues from the pCW3 family of conjugative C. perfringens plasmids specifically recognize and bind to their cognate *parC* sites, providing biochemical evidence for the biological relevance of the phylogenetic ParMRC families that were previously identified (34). DNA binding studies showed that ParR proteins interacted with sequences within a centromeric *parC* site from the same ParMRC family but could not interact with a noncognate *parC* site from a different ParMRC family. We also demonstrated that ParR proteins can bind to noncognate *parC* sites from the same ParMRC family. These findings are consistent with our previous phenotypic analysis of ParMRC-encoding plasmids in C. perfringens, where plasmids from the same partitioning family were unable to be maintained in a single C. perfringens isolate in the absence of selection (36). These combined data provide clear experimental evidence that variation in the ParMRC partitioning systems represents a major molecular mechanism by which native C. perfringens isolates can maintain multiple closely related plasmids in the same cell.

All ParR proteins characterized to date bind to directly repeated sequences; however, the repeats they interact with vary between plasmid systems. For example, ParR from the E. coli plasmid R1 requires a minimum of two 11-bp repeats for binding (11), ParR from pB171 (E. coli) binds two 10-bp direct repeats upstream of *parM* (39), and ParR from the Staphylococcus aureus plasmid pSK41 binds to 20-bp repeats (10).

The direct repeats in the C. perfringens *parC* sites differ substantially between families with respect to both their nucleotide sequence and their spacing within the centromere. ParR_C_ binding correlated with four 17-bp direct repeats within the *parC_C_* region. These repeat structures are conserved between *parC_C_* regions of different plasmids, supporting the assertion that ParR is able to recognize and bind to these sites. In contrast, the ParR_B_ and ParR_D_ binding sites were more difficult to delineate because there were multiple direct and inverted repeat structures within the *parC_B_* and *parC_D_* regions.

Our findings support the hypothesis that the inability of ParR proteins to discriminate between closely related *parC* sites is responsible for previously observed ParMRC-mediated plasmid incompatibility (36). The consequence would be the incorrect linkage of two heterologous plasmids, eventually leaving distinct populations of daughter cells each containing only one of these plasmids (14, 17, 18, 40). Although the heterologous pairing model is not favored for type I partitioning-mediated incompatibility (16, 18), there is evidence that suggests that this model could explain ParMRC-based plasmid incompatibility. For example, ParR from R1 is capable of linking replicons before partitioning and promiscuous binding of ParR from pB171 is responsible for plasmid incompatibility (8, 20).

In this study, we have demonstrated that the interaction between ParR and *parC* is important for plasmid incompatibility; however, there is a second key recognition step in the partition reaction between the filament-forming protein ParM and the ParR-*parC* complex. ParM falls into the same phylogenetic groups as ParR and *parC*; therefore, it is likely that ParM has a specificity profile similar to that of ParR and *parC*. We postulate that ParM will interact with ParR-*parC* complexes from the same family but will not recognize noncognate ParR-*parC* complexes from other families. Further studies will aim to characterize the interaction between ParM and ParR in C. perfringens and determine whether this recognition step follows a pattern similar to that of the ParR-*parC* interactions outlined in this study.

For technical reasons already outlined, the AUC experiments were conducted using high concentrations (25 μM) of ParR_C_(pCW3). Analysis of our sedimentation velocity data showed that ParR_C_(pCW3) formed a tetrameric complex in solution; however, the concentration of ParR_C_(pCW3) used (25 μM) is unlikely to reflect a physiologically relevant level of ParR protein within the cell. Dissection of the SPR binding data between ParR_C_(pCW3) and *parC_C_*(pCW3) fragments that contain a predicted binding site suggested a 2:1 association of ParR to *parC*. Further experiments are required to confirm the oligomeric state of ParR_C_(pCW3). Furthermore, upon the addition of *parC_C_* fragments, a higher sedimentation coefficient was observed. At this concentration, ParR_C_(pCW3) interacted with the *parC_C_* C9 fragment, despite the absence of the 17-bp direct repeat, suggesting that at high concentrations, ParR_C_(pCW3) is capable of binding DNA nonspecifically. EMSA confirmed that ParR_C_(pCW3) binding to *parC_C_* (C9) was nonspecific and that ParR_C_(pCW3) binding to *parC_C_* (C5) was a specific interaction at lower concentrations (1 μM).

These data are consistent with previous structural studies of ParR proteins from pSK41 and pB171 (9, 10), which form tight dimers in solution and bind cooperatively to the DNA major groove within the *parC* centromere (5, 8–11). Once bound to *parC*, ParR forms a segrosome, where contacts between each ParR dimer are made, ultimately resulting in the formation of a dimer of dimers.

Replicon coevolution appears to be widespread in C. perfringens: different isolates often carry closely related plasmids with different ParMRC partitioning systems (22, 24, 28). For example, the avian necrotic enteritis strain EHE-NE18 has three plasmids that have similar replication proteins but different ParMRC system families (ParMRC_A_, ParMRC_B_, and ParMRC_C_) (24). Based on the ParR_B_, ParR_C_, and ParR_D_ binding data reported here, and the previous genetic studies (36), it is concluded that to ensure that each plasmid is segregated independently, these ParMRC systems have coevolved to carry different partition specificities.

The evolution of multiple ParMRC partition specificities in C. perfringens cells is reminiscent of the evolution of independent ParABS systems in Burkholderia cenocepacia. The pathogenic B. cenocepacia strain J2315 maintains three chromosomes and a large, low-copy-number plasmid (41). The type I (ParABS) partitioning systems of these replicons have coevolved to become distinct so that each replicon is partitioned independently (41–44). Likewise, Rhizobium leguminosarum bv. trifolii RepB (ParB homologue) proteins discriminate between similar *parS* centromeres to independently segregate and maintain a chromosome in addition to four plasmids (45). Unlike B. cenocepacia and R. leguminosarum, where the selection pressure to maintain multiple chromosomes and plasmids seems to have driven the coevolution of separate partition specificities, the selective pressure that has resulted in the generation of so many *parMRC* alleles in these conjugative C. perfringens plasmids remains unclear. One explanation may be that the ParMRC systems act as a means of competitive exclusion. It can be envisioned that upon entry into a new cell via conjugation, pCW3-like plasmids could displace resident plasmids that encode similar partitioning systems, thereby generating two distinct bacterial subpopulations, each carrying a single plasmid. In addition, the plasmid-borne toxin and antibiotic resistance genes may result in the positive selection of these plasmids in certain environmental niches, providing a selective advantage for the host cell if it can maintain these closely related plasmids. There is most certainly more complexity involved in the incompatibility phenotype in C. perfringens, since other factors, such as the timing of plasmid replication, the plasmid copy number, and plasmid replication initiation and regulatory proteins, may play at least some role in determining whether two replicons are incompatible or are maintained in the same cell, as in other bacteria (14, 15, 18, 46).

In conclusion, we have shown that interaction between the ParMRC partitioning components ParR and *parC* occurs only between members of the same phylogenetic family. These results provide biochemical insight into the basis of C. perfringens plasmid incompatibility and explain how multiple plasmids with similar replicons can be maintained within a single C. perfringens isolate.

## MATERIALS AND METHODS

### Plasmids, bacterial strains, and culture conditions.

All C. perfringens strains, Escherichia coli strains, and plasmids used in this study are listed in Table 1. All E. coli strains were grown on 2× yeast-tryptone (2YT) agar supplemented with 100 μg/mL of ampicillin and incubated at 37°C overnight. E. coli expression strains were grown in either 2YT broth or autoinduction medium (AIM) (47, 48).

**TABLE 1:** Bacterial strains and plasmids used in this study

Strain or plasmid	Description^*a*^	Reference or source
C. perfringens		
CN1020	Type D isolate carrying the *etx* gene on pJIR3118	53
JGS1987	Type E isolate carrying *iap/ibp*, *cpe*, *lam*, and *cpb2* toxin genes	GenBank accession no. ABDW00000000
JIR4195	JIR325(pCW3) Tc^r^	53
CN4003	Type D isolate carrying *etx*, *cpe*, *cpb2*, and *lam* toxin genes	54
E. coli		
BL21(DE3)	*fhuA2 [lon] ompT gal* (*λ DE3*) *[dcm] ΔhsdSλ DE3 = λ sBamHIo ΔEcoRI-B int*::(*lacI*::*PlacUV5*::*T7 gene1*) *i21 Δnin5*	New England Biolabs
C41(DE3)	BL21(DE3) derivative	55
C43(DE3)	BL21(DE3) derivative	55
Plasmid		
pCW3	Isolated from CW92; 47 kb; Tc^r^; *parMRC_C_*(pCW3)	56
pET22b(+)	T7 promoter expression vector; IPTG inducible; adds C-terminal His_6_ tag; Amp^r^	Novagen
pJGS1987B	Plasmid from JGS1987; carries *parMRC_B_*(pJGS1987B)	GenBank accession no. ABDW01000017
pJGS1987C	Plasmid from JGS1987; carries *parMRC_C_*(pJGS1987C)	GenBank accession no. ABDW01000012
pJIR3118	48-kb *etx*-bearing plasmid from CN1020; carries *parMRC_D_*(pJIR3118)	53
pJIR4165	CPE-encoding plasmid isolated from CN4003 (100 kb); carries *parMRC_B_*(pJIR4165)	V. Adams, D. Lyras and J. I. Rood, unpublished data
pJIR4519	pET22b(+)ΩNdeI/XhoI *parR_C_* from pCW3	This study
pJIR4767	pET22b(+)ΩNdeI/XhoI *parR_B_* from pJGS1987B	This study
pJIR4768	pET22b(+)ΩNdeI/XhoI *parR_C_* from pGS1987C	This study
pJIR4769	pET22b(+)ΩNdeI/XhoI *parR_D_* from pJGS1987D	This study
pJIR4773	pET22b(+)ΩNdeI/XhoI *parR_B_* from pJIR4165	This study
pJIR4820	pET22b(+)ΩNdeI/XhoI *parR_D_* from pJIR3118	This study

a Tc^r^, tetracycline resistant; Amp^r^, ampicillin resistant. CPE, C. perfringens enterotoxin; *etx*, epsilon-toxin gene; *lam*, lambda toxin gene; *iap* and *ibp*, iota toxin genes; *cpb2*, beta2 toxin gene.

### Construction of ParR expression vectors.

The *parR_C_* gene from pCW3 was codon optimized for expression in E. coli, synthesized by GenScript, and cloned into the EcoRV site of pUC57-Kan. Codon-optimized *parR_C_* then was subcloned into the NdeI/XhoI sites of pET22b(+). *parR_D_*(pJIR3118) was PCR amplified from CN1020 genomic DNA (gDNA) isolated as described previously (49) and cloned into the NdeI/Xhol site of pET22b(+) for expression. *parR_B_*(pJIR4165), *parR_B_*(pJGS1987B), *parR_C_*(pJGS1987C), and *parR_D_*(pJGS1987D) were codon optimized and synthesized before being cloned into pET22b(+) NdeI/XhoI sites by GenScript.

### ParR expression and purification.

ParR proteins with C-terminal His_6_ tags were expressed using E. coli strain C43(DE3), C41(DE3), or BL21(DE3). The cells either were grown at 28°C in autoinduction medium for 24 h before the temperature was lowered to 22°C for 6 h or were grown in 2YT broth at 37°C to an optical density at 600 nm (OD_600_) of 0.6 and induced with the addition of 0.1 mM IPTG (isopropyl-β-d-thiogalactopyranoside) for 3 h (Table S2). Cells were lysed using a cell disrupter (Avestin) (lysis buffer: 20 mM TRIS [pH 7.9], 300 mM NaCl, 10% glycerol, 1 mg/mL of DNase I, and cOmplete protease inhibitors [Roche]), and proteins were purified (Fig. S1) using TALON resin (Clontech) and eluted with the addition of increasing concentrations of imidazole (5 mM to 200 mM) in purification buffer (20 mM Tris [pH 7.9], 300 mM NaCl, 10% glycerol) and confirmed by Western blotting. All ParR proteins were buffer exchanged into buffer A (10 mM HEPES [pH 7.4], 300 mM NaCl, 3 mM EDTA, 0.05% Tween 20, 0.02% NaN_3_) using a 3-kDa centrifugal filter (Amicon) before dilution to 0.1 μM. Independent preparations of each purified ParR protein were used as biological repeats for SPR.

10.1128/mbio.01356-22.6 TABLE S2 ParR protein properties and expression conditions. Download Table S2, DOCX file, 0.01 MB. Copyright © 2022 Watts et al. 2022 Watts et al. https://creativecommons.org/licenses/by/4.0/ This content is distributed under the terms of the Creative Commons Attribution 4.0 International license.

### Fragment array preparation for SPR experiments.

*parC* fragment arrays were constructed as previously described (37) using the reusable DNA capture technique (ReDCaT). Briefly, the *parC* regions of pCW3 (192 bp), pJIR3118 (230 bp), and pJIR4165 (262 bp) were used as templates for the Perl overlapping oligonucleotide program (POOP). POOP produced a series of overlapping forward and reverse 30-bp oligonucleotides (20-bp overlap). Reverse-strand oligonucleotides had a 20-bp 3′ sequence (5′-CCTACCCTACGTCCTCCTGC-3′) that was complementary to the ReDCaT sequence. The *parC_C_* C1 and *parC_B_* B17 mutant fragments and the *parC_D_* D3+D4 footprinting oligonucleotides were constructed as described above (the ligands used in SPR experiments are listed in Table S3). Oligonucleotides were synthesized (Integrated DNA Technologies) at a concentration of 100 μM in IDTE buffer (10 mM Tris, 0.1 mM EDTA [pH 8.0]). To construct fragments for SPR analysis, complementary oligonucleotides were mixed in a ratio of 1.2:1 (forward to reverse), annealed at 98°C for 10 min, and cooled for 30 min at room temperature. Fragments were then diluted to 0.5 nM in buffer A.

10.1128/mbio.01356-22.7 TABLE S3 *parC* oligonucleotides and ligand molecular weight and size. Download Table S3, DOCX file, 0.02 MB. Copyright © 2022 Watts et al. 2022 Watts et al. https://creativecommons.org/licenses/by/4.0/ This content is distributed under the terms of the Creative Commons Attribution 4.0 International license.

### Surface plasmon resonance.

SPR experiments were based upon the ReDCaT method as previously described (37) and conducted using the Biacore T200 system (GE Healthcare Life Sciences). All experiments were carried out on an S series Biacore sensor chip (GE Healthcare Life Sciences) with streptavidin (SA) preimmobilized to a carboxymethylated dextran matrix for capture of biotinylated interaction partners.

Prior to SPR, all four flow cells of the SA chip were washed three times with buffer containing 1 M NaCl and 50 mM NaOH. After washing and priming with buffer A, biotinylated ReDCaT linker (100 nM) (5′-biotin-GCAGGAGGACGTAGGGTAGG-3′) was immobilized to all four flow cells at 5 μL/min to a capture level of ~500 response units (RU). Subsequently, the chip was primed with buffer A, the ReDCaT complementary oligonucleotide (500 nM) was captured on flow cell 1, and *parC* ligands diluted in buffer A to a concentration of 500 nM were captured to flow cells 2 to 4 (*parC_B_*, *parC_C_*, and *parC_D_* fragments on flow cells 2, 3, and 4, respectively) to a density of approximately 200 RU under flow conditions (10 μL/min for 30 s). DNA capture levels are listed in Table S4. The first flow cell was used as a reference cell for subsequent measurements on flow cells 2 to 4. Each ParR protein [ParR_B_(pJIR4165), ParR_B_(pJGS1987B), ParR_C_(pCW3), ParR_C_(pJGS1987), and ParR_D_(pJGS1987)] was diluted to a concentration of 0.1 μM in buffer A, and ParR_D_(pJIR3118) was diluted in buffer A with 1 mg/mL of dextran to reduce nonspecific binding. Proteins were flowed through all four flow cells at 30 μL/min with 60 s of association and 60 s of dissociation. Binding stability measurements were recorded 10 s after the end of sample injection. All four flow cells of the chip were regenerated after each cycle using regeneration buffer (1 M NaCl and 50 mM NaOH) to leave only the biotinylated ReDCaT oligonucleotide. All experiments were conducted at 20°C. All SPR methods were programmed using Biacore T200 control software, and data were analyzed using Biacore evaluation software version 2.0. The ParR_C_(pCW3)-*parC_C_*(pCW3) binding stoichiometry was calculated by dividing the background subtracted RU recorded for ParR_C_ by the RU of immobilized DNA deposited on the sensor chip.

10.1128/mbio.01356-22.8 TABLE S4 Binding of ParR homologues to *parC* using surface plasmon resonance. Download Table S4, DOCX file, 0.03 MB. Copyright © 2022 Watts et al. 2022 Watts et al. https://creativecommons.org/licenses/by/4.0/ This content is distributed under the terms of the Creative Commons Attribution 4.0 International license.

### Analytical ultracentrifugation.

Sedimentation velocity experiments were performed in an Optima analytical ultracentrifuge (Beckman Coulter) equipped with UV-visible (UV-Vis) scanning optics. ParR_C_(pCW3) was prepared at a concentration of 0.5 mg/mL with and without 0.1 mg/mL of *parC_C_* DNA (fragment C5). Reference (400 μL of buffer A without Tween 20) and sample (370 μL) solutions were loaded into double-sector cells with quartz windows. These cells were mounted in an An-50 Ti 8-hole rotor. Proteins and DNA were centrifuged at 40,000 rpm at 20°C, and radial absorbance data were collected at appropriate wavelengths (~280 nm) in continuous mode every 20 s. The partial specific volume (−ν) of ParR_C_ (0.7372), buffer density (1.0119 g/mL), and buffer viscosity (0.0104 poise [P]) were determined using the program SEDNTERP (50). The −ν of *parC_C_* C5 DNA (0.5500) was determined using UltraScan III (51). Data were fitted to continuous size distribution [*c*(*s*)] and continuous mass distribution [*c*(*M*)] models using the program SEDFIT (52). All sedimentation coefficient data were normalized to standard conditions at 20°C in water (*s*_20,W_); relevant hydrodynamic properties are listed in Table S5.

10.1128/mbio.01356-22.9 TABLE S5 Hydrodynamic properties of ParR_C_(pCW3) and *parC_C_*(pCW3) C5 DNA determined by AUC experiments. Download Table S5, DOCX file, 0.01 MB. Copyright © 2022 Watts et al. 2022 Watts et al. https://creativecommons.org/licenses/by/4.0/ This content is distributed under the terms of the Creative Commons Attribution 4.0 International license.

### Electrophoretic mobility shift assay.

Target 30-bp DNA fragments were generated by annealing forward and reverse oligonucleotides. All gel shift DNA was labeled with digoxigenin-11-ddUTP (DIG) at their 3′ termini with the DIG gel shift kit (Roche) as per the manufacturer’s instructions. Gel mobility shift assays were carried out using the DIG gel shift kit, 2nd generation (Roche). Reactions testing ParR_C_(pCW3) binding included 4 μL of binding buffer [100 mM HEPES (pH 7.6), 5 mM EDTA, 50 mM (NH_4_)_2_SO_4_, 5 mM dithiothreitol (DTT), 1% (wt/vol) Tween 20, 150 mM (KCl) (Roche), 1 μg of poly[d(I-C)]], 0.1 μg of poly-l-lysine, 1 pmol of DIG-labeled target DNA, 1 pmol (1:1) or 4 pmol (1:4) of His_6_-ParR_C_(pCW3), and sterile deionized water in a total volume of 20 μL. For reactions that tested ParR_C_(pCW3) binding specificity, 150 to 200 pmol of unlabeled *parC_C_*(pCW3) (C9) or unlabeled *parC_C_*(pCW3) (C5) DNA was added to reaction mixtures containing 1 pmol of DIG-labeled *parC_C_*(pCW3) (C5) DNA and 1 pmol of His_6_-ParR_C_. Reaction mixtures were incubated for 15 min at room temperature before the addition of gel loading buffer without bromophenol blue. Reaction mixtures were loaded immediately onto 10% (wt/vol) native 1× TBE (22.3 mM Tris, 22.3 mM boric acid, 0.5 mM EDTA [pH 8.0]) polyacrylamide gels with a control lane containing gel loading buffer with bromophenol blue. Samples were separated at 173 V for 40 min and then transferred onto Nylon+ membranes (Amersham Life Science, UK) by electroblotting with 1× TBE (pH 8.0) at 100 V for 1 h. Following transfer, the membrane was UV cross-linked and chemiluminescent detection of DIG epitope was carried out as per the manufacturer’s instructions (Roche). Chemiluminescence was recorded using Bio-Rad Chemidoc+ imaging systems (Bio-Rad).

## References

[1] Gerdes K, Howard M, Szardenings F. 2010. Pushing and pulling in prokaryotic DNA segregation. Cell 141:927–942. doi:10.1016/j.cell.2010.05.033. 20550930

[2] Moller-Jensen J, Jensen RB, Lowe J, Gerdes K. 2002. Prokaryotic DNA segregation by an actin-like filament. EMBO J 21:3119–3127. doi:10.1093/emboj/cdf320. 12065424 PMC126073

[3] van den Ent F, Moller-Jensen J, Amos LA, Gerdes K, Lowe J. 2002. F-actin-like filaments formed by plasmid segregation protein ParM. EMBO J 21:6935–6943. doi:10.1093/emboj/cdf672. 12486014 PMC139093

[4] Breuner A, Jensen RB, Dam M, Pedersen S, Gerdes K. 1996. The centromere-like *parC* locus of plasmid R1. Mol Microbiol 20:581–592. doi:10.1046/j.1365-2958.1996.5351063.x. 8736537

[5] Dam M, Gerdes K. 1994. Partitioning of plasmid R1. Ten direct repeats flanking the parA promoter constitute a centromere-like partition site *parC*, that expresses incompatibility. J Mol Biol 236:1289–1298. doi:10.1016/0022-2836(94)90058-2. 8126720

[6] Gerdes K, Rasmussen PB, Molin S. 1986. Unique type of plasmid maintenance function: postsegregational killing of plasmid-free cells. Proc Natl Acad Sci USA 83:3116–3120. doi:10.1073/pnas.83.10.3116. 3517851 PMC323463

[7] Jensen RB, Dam M, Gerdes K. 1994. Partitioning of plasmid R1. The *parA* operon is autoregulated by ParR and its transcription is highly stimulated by a downstream activating element. J Mol Biol 236:1299–1309. doi:10.1016/0022-2836(94)90059-0. 8126721

[8] Jensen RB, Lurz R, Gerdes K. 1998. Mechanism of DNA segregation in prokaryotes: replicon pairing by *parC* of plasmid R1. Proc Natl Acad Sci USA 95:8550–8555. doi:10.1073/pnas.95.15.8550. 9671715 PMC21113

[9] Moller-Jensen J, Ringgaard S, Mercogliano CP, Gerdes K, Lowe J. 2007. Structural analysis of the ParR/*parC* plasmid partition complex. EMBO J 26:4413–4422. doi:10.1038/sj.emboj.7601864. 17898804 PMC2034672

[10] Schumacher MA, Glover TC, Brzoska AJ, Jensen SO, Dunham TD, Skurray RA, Firth N. 2007. Segrosome structure revealed by a complex of ParR with centromere DNA. Nature 450:1268–1271. doi:10.1038/nature06392. 18097417

[11] Moller-Jensen J, Borch J, Dam M, Jensen RB, Roepstorff P, Gerdes K. 2003. Bacterial mitosis: ParM of plasmid R1 moves plasmid DNA by an actin-like insertional polymerization mechanism. Mol Cell 12:1477–1487. doi:10.1016/s1097-2765(03)00451-9. 14690601

[12] Gayathri P, Fujii T, Moller-Jensen J, van den Ent F, Namba K, Lowe J. 2012. A bipolar spindle of antiparallel ParM filaments drives bacterial plasmid segregation. Science 338:1334–1337. doi:10.1126/science.1229091. 23112295 PMC3694215

[13] Bharat TA, Murshudov GN, Sachse C, Lowe J. 2015. Structures of actin-like ParM filaments show architecture of plasmid-segregating spindles. Nature 523:106–110. doi:10.1038/nature14356. 25915019 PMC4493928

[14] Novick RP. 1987. Plasmid incompatibility. Microbiol Rev 51:381–395. doi:10.1128/mr.51.4.381-395.1987. 3325793 PMC373122

[15] Ebersbach G, Sherratt DJ, Gerdes K. 2005. Partition-associated incompatibility caused by random assortment of pure plasmid clusters. Mol Microbiol 56:1430–1440. doi:10.1111/j.1365-2958.2005.04643.x. 15916596

[16] Bouet JY, Rech J, Egloff S, Biek DP, Lane D. 2005. Probing plasmid partition with centromere-based incompatibility. Mol Microbiol 55:511–525. doi:10.1111/j.1365-2958.2004.04396.x. 15659167

[17] Funnell BE. 2005. Partition-mediated plasmid pairing. Plasmid 53:119–125. doi:10.1016/j.plasmid.2004.12.009. 15737399

[18] Bouet JY, Nordstrom K, Lane D. 2007. Plasmid partition and incompatibility—the focus shifts. Mol Microbiol 65:1405–1414. doi:10.1111/j.1365-2958.2007.05882.x. 17714446

[19] Bouet JY, Funnell BE. 12 June 2019. Plasmid localization and partition in Enterobacteriaceae. EcoSal Plus 2019 doi:10.1128/ecosalplus.ESP-0003-2019. PMC11573283 31187729

[20] Hyland EM, Wallace EW, Murray AW. 2014. A model for the evolution of biological specificity: a cross-reacting DNA-binding protein causes plasmid incompatibility. J Bacteriol 196:3002–3011. doi:10.1128/JB.01811-14. 24914185 PMC4135640

[21] Uzal FA, Freedman JC, Shrestha A, Theoret JR, Garcia J, Awad MM, Adams V, Moore RJ, Rood JI, McClane BA. 2014. Towards an understanding of the role of *Clostridium perfringens* toxins in human and animal disease. Future Microbiol 9:361–377. doi:10.2217/fmb.13.168. 24762309 PMC4155746

[22] Li J, Adams V, Bannam TL, Miyamoto K, Garcia JP, Uzal FA, Rood JI, McClane BA. 2013. Toxin plasmids of *Clostridium perfringens*. Microbiol Mol Biol Rev 77:208–233. doi:10.1128/MMBR.00062-12. 23699255 PMC3668675

[23] Bannam TL, Teng WL, Bulach D, Lyras D, Rood JI. 2006. Functional identification of conjugation and replication regions of the tetracycline resistance plasmid pCW3 from *Clostridium perfringens*. J Bacteriol 188:4942–4951. doi:10.1128/JB.00298-06. 16788202 PMC1483020

[24] Bannam TL, Yan XX, Harrison PF, Seemann T, Keyburn AL, Stubenrauch C, Weeramantri LH, Cheung JK, McClane BA, Boyce JD, Moore RJ, Rood JI. 2011. Necrotic enteritis-derived *Clostridium perfringens* strain with three closely related independently conjugative toxin and antibiotic resistance plasmids. mBio 2:e00190-11. doi:10.1128/mBio.00190-11. 21954306 PMC3181468

[25] Han X, Du XD, Southey L, Bulach DM, Seemann T, Yan XX, Bannam TL, Rood JI. 2015. Functional analysis of a bacitracin resistance determinant located on ICE*Cp1*, a novel Tn*916*-like element from a conjugative plasmid in *Clostridium perfringens*. Antimicrob Agents Chemother 59:6855–6865. doi:10.1128/AAC.01643-15. 26282424 PMC4604367

[26] Miyamoto K, Fisher DJ, Li J, Sayeed S, Akimoto S, McClane BA. 2006. Complete sequencing and diversity analysis of the enterotoxin-encoding plasmids in *Clostridium perfringens* type A non-food-borne human gastrointestinal disease isolates. J Bacteriol 188:1585–1598. doi:10.1128/JB.188.4.1585-1598.2006. 16452442 PMC1367241

[27] Miyamoto K, Li J, Sayeed S, Akimoto S, McClane BA. 2008. Sequencing and diversity analyses reveal extensive similarities between some epsilon-toxin-encoding plasmids and the pCPF5603 *Clostridium perfringens* enterotoxin plasmid. J Bacteriol 190:7178–7188. doi:10.1128/JB.00939-08. 18776010 PMC2580689

[28] Parreira VR, Costa M, Eikmeyer F, Blom J, Prescott JF. 2012. Sequence of two plasmids from *Clostridium perfringens* chicken necrotic enteritis isolates and comparison with *C. perfringens* conjugative plasmids. PLoS One 7:e49753. doi:10.1371/journal.pone.0049753. 23189158 PMC3506638

[29] Revitt-Mills SA, Vidor CJ, Watts TD, Lyras D, Rood JI, Adams V. 2019. Virulence plasmids of the pathogenic clostridia. Microbiol Spectr 7:7.3.7. doi:10.1128/microbiolspec.GPP3-0034-2018. PMC11257192 31111816

[30] Wisniewski JA, Rood JI. 2017. The Tcp conjugation system of *Clostridium perfringens*. Plasmid 91:28–36. doi:10.1016/j.plasmid.2017.03.001. 28286218

[31] Traore DAK, Wisniewski JA, Flanigan SF, Conroy PJ, Panjikar S, Mok YF, Lao C, Griffin MDW, Adams V, Rood JI, Whisstock JC. 2018. Crystal structure of TcpK in complex with *oriT* DNA of the antibiotic resistance plasmid pCW3. Nat Commun 9:3732. doi:10.1038/s41467-018-06096-2. 30213934 PMC6137059

[32] Revitt-Mills S, Lao C, Archambault M, Lyras D, Rood JI, Adams V. 2020. The Tcp plasmids of *Clostridium perfringens* require the *resP* gene to ensure stable inheritance. Plasmid 107:102461. doi:10.1016/j.plasmid.2019.102461. 31715189

[33] Revitt-Mills SA, Watts TD, Lyras D, Adams V, Rood JI. 2021. The ever-expanding tcp conjugation locus of pCW3 from *Clostridium perfringens*. Plasmid 113:102516. doi:10.1016/j.plasmid.2020.102516. 32526229

[34] Adams V, Watts TD, Bulach DM, Lyras D, Rood JI. 2015. Plasmid partitioning systems of conjugative plasmids from *Clostridium perfringens*. Plasmid 80:90–96. doi:10.1016/j.plasmid.2015.04.004. 25929175

[35] Chen S, Larsson M, Robinson RC, Chen SL. 2017. Direct and convenient measurement of plasmid stability in lab and clinical isolates of *E. coli*. Sci Rep 7:4788. doi:10.1038/s41598-017-05219-x. 28684862 PMC5500522

[36] Watts TD, Johanesen PA, Lyras D, Rood JI, Adams V. 2017. Evidence that compatibility of closely related replicons in *Clostridium perfringens* depends on linkage to *parMRC*-like partitioning systems of different subfamilies. Plasmid 91:68–75. doi:10.1016/j.plasmid.2017.03.008. 28390955

[37] Stevenson CE, Assaad A, Chandra G, Le TB, Greive SJ, Bibb MJ, Lawson DM. 2013. Investigation of DNA sequence recognition by a streptomycete MarR family transcriptional regulator through surface plasmon resonance and X-ray crystallography. Nucleic Acids Res 41:7009–7022. doi:10.1093/nar/gkt523. 23748564 PMC3737563

[38] McWilliam H, Li W, Uludag M, Squizzato S, Park YM, Buso N, Cowley AP, Lopez R. 2013. Analysis tool Web Services from the EMBL-EBI. Nucleic Acids Res 41:W597–W600. doi:10.1093/nar/gkt376. 23671338 PMC3692137

[39] Ringgaard S, Ebersbach G, Borch J, Gerdes K. 2007. Regulatory cross-talk in the double par locus of plasmid pB171. J Biol Chem 282:3134–3145. doi:10.1074/jbc.M609092200. 17092933

[40] Austin S, Nordstrom K. 1990. Partition-mediated incompatibility of bacterial plasmids. Cell 60:351–354. doi:10.1016/0092-8674(90)90584-2. 2406018

[41] Dubarry N, Pasta F, Lane D. 2006. ParABS systems of the four replicons of *Burkholderia cenocepacia*: new chromosome centromeres confer partition specificity. J Bacteriol 188:1489–1496. doi:10.1128/JB.188.4.1489-1496.2006. 16452432 PMC1367244

[42] Passot FM, Calderon V, Fichant G, Lane D, Pasta F. 2012. Centromere binding and evolution of chromosomal partition systems in the Burkholderiales. J Bacteriol 194:3426–3436. doi:10.1128/JB.00041-12. 22522899 PMC3434744

[43] Du WL, Dubarry N, Passot FM, Kamgoue A, Murray H, Lane D, Pasta F. 2016. Orderly replication and segregation of the four replicons of *Burkholderia cenocepacia* J2315. PLoS Genet 12:e1006172. doi:10.1371/journal.pgen.1006172. 27428258 PMC4948915

[44] Pillet F, Passot FM, Pasta F, Anton Leberre V, Bouet JY. 2017. Analysis of ParB-centromere interactions by multiplex SPR imaging reveals specific patterns for binding ParB in six centromeres of Burkholderiales chromosomes and plasmids. PLoS One 12:e0177056. doi:10.1371/journal.pone.0177056. 28562673 PMC5450999

[45] Koper P, Żebracki K, Marczak M, Skorupska A, Mazur A. 2016. RepB proteins of the multipartite *Rhizobium leguminosarum* bv. *trifolii* genome discriminate between centromere-like *parS* sequences for plasmid segregational stability. Mol Microbiol 102:446–466. doi:10.1111/mmi.13472. 27480612

[46] Diaz R, Rech J, Bouet JY. 2015. Imaging centromere-based incompatibilities: insights into the mechanism of incompatibility mediated by low-copy number plasmids. Plasmid 80:54–62. doi:10.1016/j.plasmid.2015.03.007. 25889267

[47] Studier FW. 2005. Protein production by auto-induction in high density shaking cultures. Protein Expr Purif 41:207–234. doi:10.1016/j.pep.2005.01.016. 15915565

[48] Studier FW. 2014. Stable expression clones and auto-induction for protein production in *E. coli*. Methods Mol Biol 1091:17–32. doi:10.1007/978-1-62703-691-7_2. 24203322

[49] O’Connor JR, Lyras D, Farrow KA, Adams V, Powell DR, Hinds J, Cheung JK, Rood JI. 2006. Construction and analysis of chromosomal *Clostridium difficile* mutants. Mol Microbiol 61:1335–1351. doi:10.1111/j.1365-2958.2006.05315.x. 16925561

[50] Laue TM. 1992. Computer-aided interpretation of analytical sedimentation data for proteins, p 90–125. *In* Harding SE, Rowe AJ, Horton JC (ed), Analytical ultracentrifugation in biochemistry and polymer science. Royal Society of Chemistry, London, United Kingdom.

[51] Demeler B. 2005. UltraScan—a comprehensive data analysis software package for analytical ultracentrifugation experiments, p 210–230. *In* Scott DJ, Harding SE, Rowe AJ (ed), Analytical ultracentrifugation: techniques and methods. Royal Society of Chemistry, London, United Kingdom.

[52] Schuck P. 2000. Size-distribution analysis of macromolecules by sedimentation velocity ultracentrifugation and lamm equation modeling. Biophys J 78:1606–1619. doi:10.1016/S0006-3495(00)76713-0. 10692345 PMC1300758

[53] Hughes ML, Poon R, Adams V, Sayeed S, Saputo J, Uzal FA, McClane BA, Rood JI. 2007. Epsilon-toxin plasmids of *Clostridium perfringens* type D are conjugative. J Bacteriol 189:7531–7538. doi:10.1128/JB.00767-07. 17720791 PMC2168747

[54] Sayeed S, Fernandez-Miyakawa ME, Fisher DJ, Adams V, Poon R, Rood JI, Uzal FA, McClane BA. 2005. Epsilon-toxin is required for most *Clostridium perfringens* type D vegetative culture supernatants to cause lethality in the mouse intravenous injection model. Infect Immun 73:7413–7421. doi:10.1128/IAI.73.11.7413-7421.2005. 16239541 PMC1273886

[55] Miroux B, Walker JE. 1996. Over-production of proteins in *Escherichia coli*: mutant hosts that allow synthesis of some membrane proteins and globular proteins at high levels. J Mol Biol 260:289–298. doi:10.1006/jmbi.1996.0399. 8757792

[56] Rood JI, Scott VN, Duncan CL. 1978. Identification of a transferable tetracycline resistance plasmid (pCW3) from *Clostridium perfringens*. Plasmid 1:563–570. doi:10.1016/0147-619x(78)90013-6. 219433

