# Supplementary material for: The Specificity of ParR Binding Determines the Incompatibility of Conjugative Plasmids in Clostridium perfringens
Source: mBio. 2022 Jun 21;13(4):e01356-22. doi: 10.1128/mbio.01356-22 (PMC9426499; doi:10.1128/mbio.01356-22)

**Supplementary Figure 1.** Purified recombinant ParR proteins. ParR proteins were purified and analysed using a 15% polyacrylamide gel stained with Coomassie brilliant blue. Legend: 1 BioRad protein standards, 2 ParR_B_(pJIR4165), 3 ParR_B_(pJGS1987B), 4 ParR_C_(pCW3), 5 ParR_C_(pJGS1987C), 6 ParR_D_(pJGS1987D), 7 ParR_D_(pJIR3118).


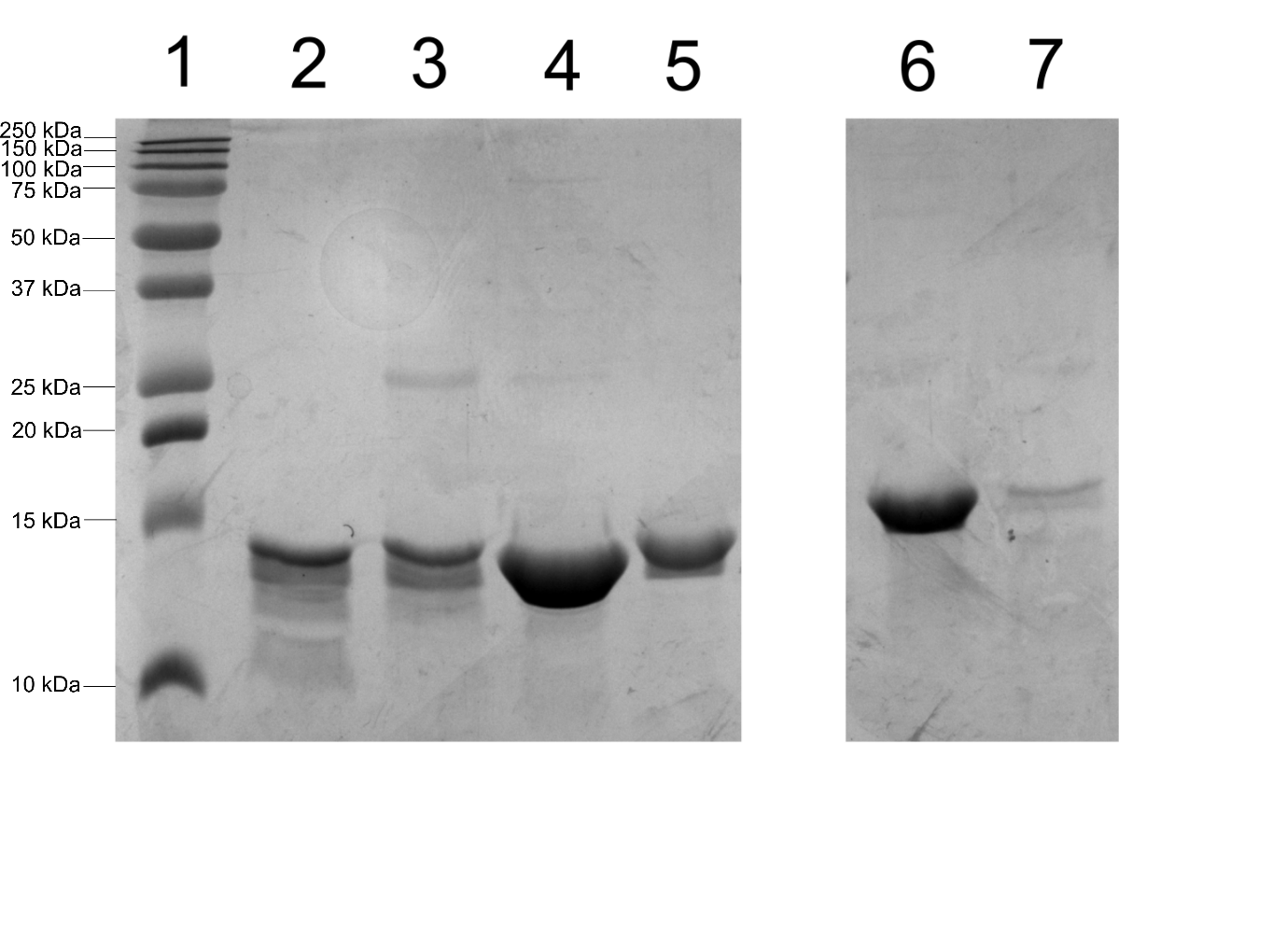

Supplement: FIG S1 [file mbio.01356-22-s0007.docx]
