# Supplementary material for: The Specificity of ParR Binding Determines the Incompatibility of Conjugative Plasmids in Clostridium perfringens
Source: mBio. 2022 Jun 21;13(4):e01356-22. doi: 10.1128/mbio.01356-22 (PMC9426499; doi:10.1128/mbio.01356-22)
Supplement: FIG S2 [file mbio.01356-22-s0008.docx]

**Supplementary Figure 2. Binding of ParR_C_(pCW3) with *parC_C_*(pCW3) fragments.**

**Fig S2A. Analytical Ultra-Centrifugation shows that at high concentrations ParR_C_(pCW3) interacts non-specifically with *parC_C_*(pCW3) fragments.** Analytical Ultra-Centrifugation of ParR_C_(pCW3) mixed with *parC_C_*(pCW3) fragments either containing the direct repeat binding site (C5 shown in red) or without the direct repeat binding site (C9 shown in blue). First panel is *parC_C_*(pCW3) fragments alone, middle panel shows ParR_C_(pCW3) + *parC_C_*(pCW3) in 2:1 ratio, and final panel shows ParR_C_(pCW3) + *parC_C_*(pCW3). The shift in S_20_,_W_ shows that ParR interacts with both *parC* fragments indicating ParR_C_ binds non-specifically at high concentrations.


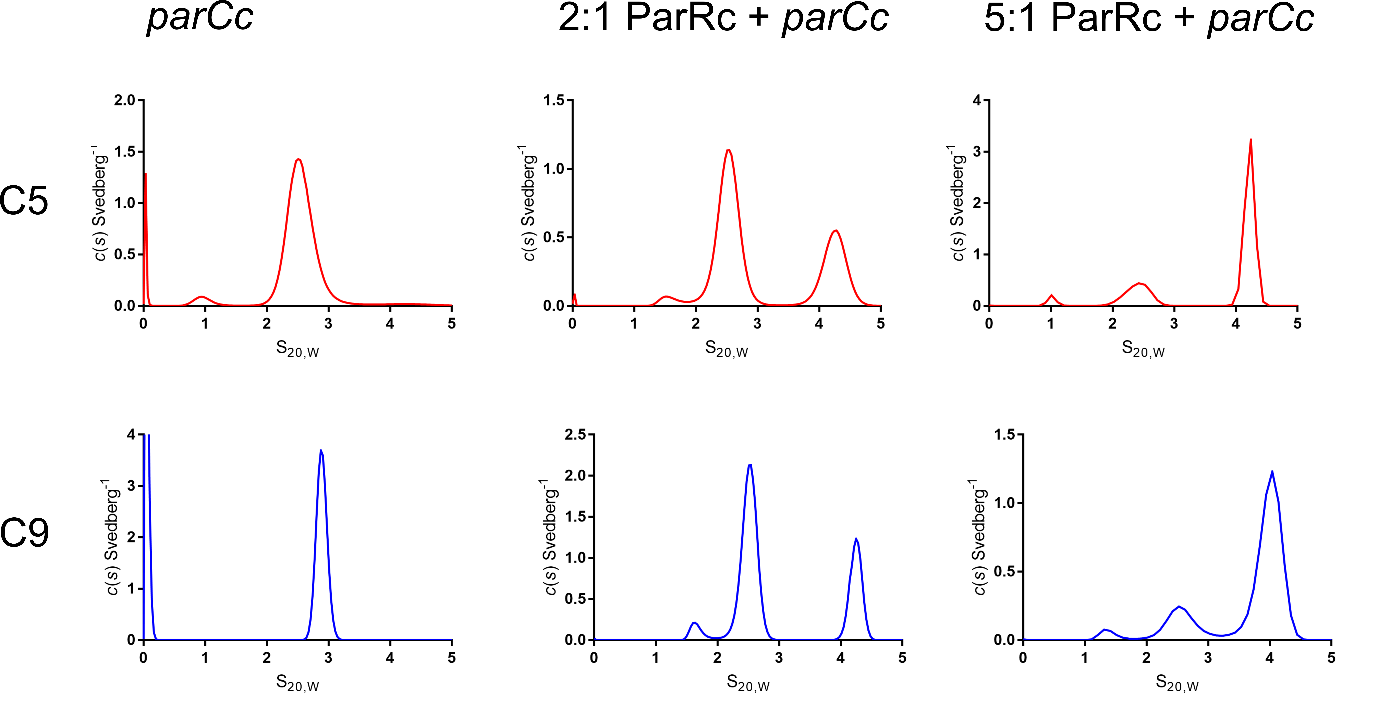


**Fig S2B. Electrophoretic mobility shift assay shows ParRC binds specifically to *parC*_C_ fragments containing 17 bp direct repeat.** F= free DNA, C = ParR-*parC_C_* (C5) complex, C5 unlabelled = unlabelled *parC_C_* (C9) in excess (200×) non-specific competitor, unlabelled C5 = unlabelled *parC_C_* (C5) in excess (200×), specific competitor. **1** No protein control, **2** labelled *parC_C_* (C5) fragment + ParR_C_(pCW3) (1 pmol *parC_C_* (C5): 1 pmol protein), **3** labelled *parC_C_* (C5) fragment + ParR_C_(pCW3) (1 pmol *parC_C_* (C5): 4 pmol protein), **4** labelled *parC_C_* (C5) + unlabelled C9, **5** labelled *parC_C_* (C5) + NSC (C9) + ParR_C_(pCW3) (1 pmol *parC_C_* (C5): 1 pmol protein), **6** labelled *parC_C_* (C5) + NSC (C9) + ParR_C_(pCW3) (1 pmol *parC_C_* (C5): 4 pmol protein). **7** No protein, **8** labelled *parC_C_* (C5) fragment + ParR_C_(pCW3) (1 pmol *parC_C_* (C5): 1 pmol protein) **9** ParR_C_(pCW3) **+** labelled *parC_C_* (C5) + unlabelled C5.

**
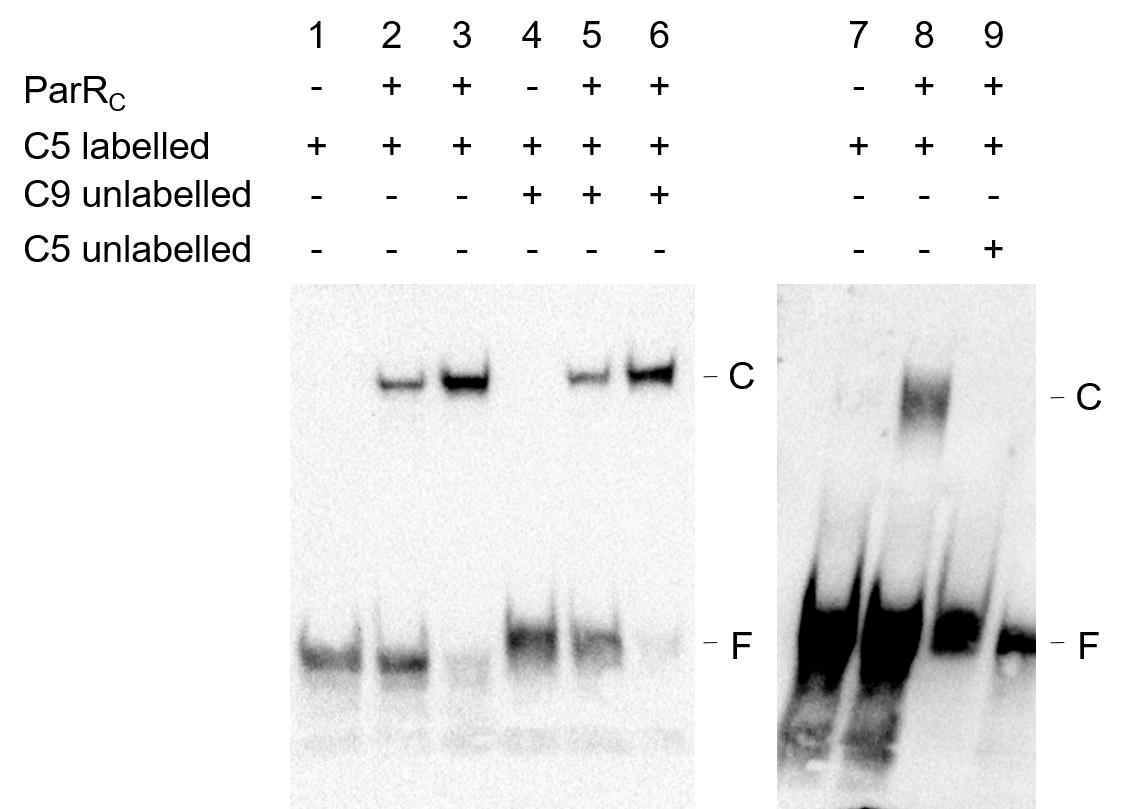
**
