# Supplementary material for: The Specificity of ParR Binding Determines the Incompatibility of Conjugative Plasmids in Clostridium perfringens
Source: mBio. 2022 Jun 21;13(4):e01356-22. doi: 10.1128/mbio.01356-22 (PMC9426499; doi:10.1128/mbio.01356-22)
Supplement: TABLE S6 [file mbio.01356-22-s0006.docx]

**Supplementary Table 6. ParR_C_-*parC_C_* (pCW3) predicted binding site stoichiometry as detected via SPR**

| ***parC_C_* fragments** | **ParR RU** | **DNA RU** | **Ratio** | **Approximate stoichiometry (ParR:*parC*)** |
| --- | --- | --- | --- | --- |
| *parC_C_* (C1) + ParR_C_ (pCW3) | 119.5 | 256.3 | 2.14 | ~2:1 |
| *parC_C_* (C5) + ParR_C_ (pCW3) | 249.5 | 123.7 | 2.02 | ~2:1 |
| *parC_C_* (C6) + ParR_C_ (pCW3) | 149.1 | 282.2 | 1.89 | ~2:1 |
| *parC_C_* (C11) + ParR_C_ (pCW3) | 148.7 | 154.7 | 1.04 | ~1:1 |
| *parC_C_* (C12) + ParR_C_ (pCW3) | 187.8 | 348.4 | 1.86 | ~2:1 |
| *parC_C_* (C15) + ParR_C_ (pCW3) | 169 | 217 | 1.28 | ~1:1 |
| *parC_C_* (C16) + ParR_C_ (pCW3) | 157.1 | 311.2 | 1.98 | ~2:1 |
