# Supplementary material for: The Specificity of ParR Binding Determines the Incompatibility of Conjugative Plasmids in Clostridium perfringens
Source: mBio. 2022 Jun 21;13(4):e01356-22. doi: 10.1128/mbio.01356-22 (PMC9426499; doi:10.1128/mbio.01356-22)
Supplement: TABLE S1 [file mbio.01356-22-s0001.docx]

**Supplementary Table 1. Amino acid sequence identity matrix of ParR homologues and nucleotide sequence identity matrix of *parC* regions**

| **A. ParR amino acid sequence identity matrix** | | | | | | |
| --- | --- | --- | --- | --- | --- | --- |
|  | ParR_C_  (pCW3) | ParR_C_  (pJGS1987C) | ParR_B_  (pJIR4165) | ParR_B_  (pJGS1987B) | ParR_D_  (pJIR3118) | ParR_D_  (pJGS1987D) |
| ParR_C_  (pCW3) | 100% |  |  |  |  |  |
| ParR_C_  (pJGS1987C) | 95% | 100% |  |  |  |  |
| ParR_B_  (pJIR4165) | 11% | 13% | 100% |  |  |  |
| ParR_B_  (pJGS1987B) | 11% | 13% | 96% | 100% |  |  |
| ParR_D_  (pJIR3118) | 26% | 26% | 24% | 24% | 100% |  |
| ParR_D_  (pJGS1987D) | 24% | 24% | 25% | 24% | 95% | 100% |
| **B. *parC* nucleotide sequence identity matrix** | | | | | | |
|  | *parC*_B_  (pJIR4165)^a^ | *parC_B_*  (pJGS1987B) | *parC*_C_  (pCW3)^a^ | *parC_C_*  (pJGS1987C) | *parC*_D_  (pJIR3118)^a^ | *parC_D_*  (pJGS1987D) |
| *parC_B_*  (pJIR4165)^a^ | 100% |  |  |  |  |  |
| *parC_B_*  (pJGS1987B) | 85% | 100% |  |  |  |  |
| *parC_C_*  (pCW3)^a^ | 45% | 43% | 100% |  |  |  |
| *parC_C_*  (pJGS1987C) | 48% | 47% | 91% | 100% |  |  |
| *parC_D_*  (pJIR3118)^a^ | 48% | 50% | 47% | 50% | 100% |  |
| *parC_D_*  (pJGS1987D) | 53% | 55% | 54% | 54% | 82% | 100% |

Percentage nt and aa similarity determined using Clustal Omega multiple sequence alignment (McWilliam et al., 2013)

^a^*parC* regions were used to generate fragment arrays for surface plasmon resonance experiments
