# Supplementary material for: The Specificity of ParR Binding Determines the Incompatibility of Conjugative Plasmids in Clostridium perfringens
Source: mBio. 2022 Jun 21;13(4):e01356-22. doi: 10.1128/mbio.01356-22 (PMC9426499; doi:10.1128/mbio.01356-22)

**Supplementary Figure 3.** **Conserved repeats in *parC* regions.** Intra-family alignments of each *parC* region. **A)** shows a nucleotide sequence alignment between *parC_B_*(pJIR4165) and *parC_B_*(pJGS1987B), putative binding sites direct repeats are shown in green **B)** nucleotide sequence alignment between *parC_C_*(pCW3) and *parC_C_*(pJGS1987C), conserved direct repeats - pink. **C)** nucleotide sequence alignment of *parC_D_*(pJIR3118) and *parC_D_*(pJGS1987D), direct repeats – blue.


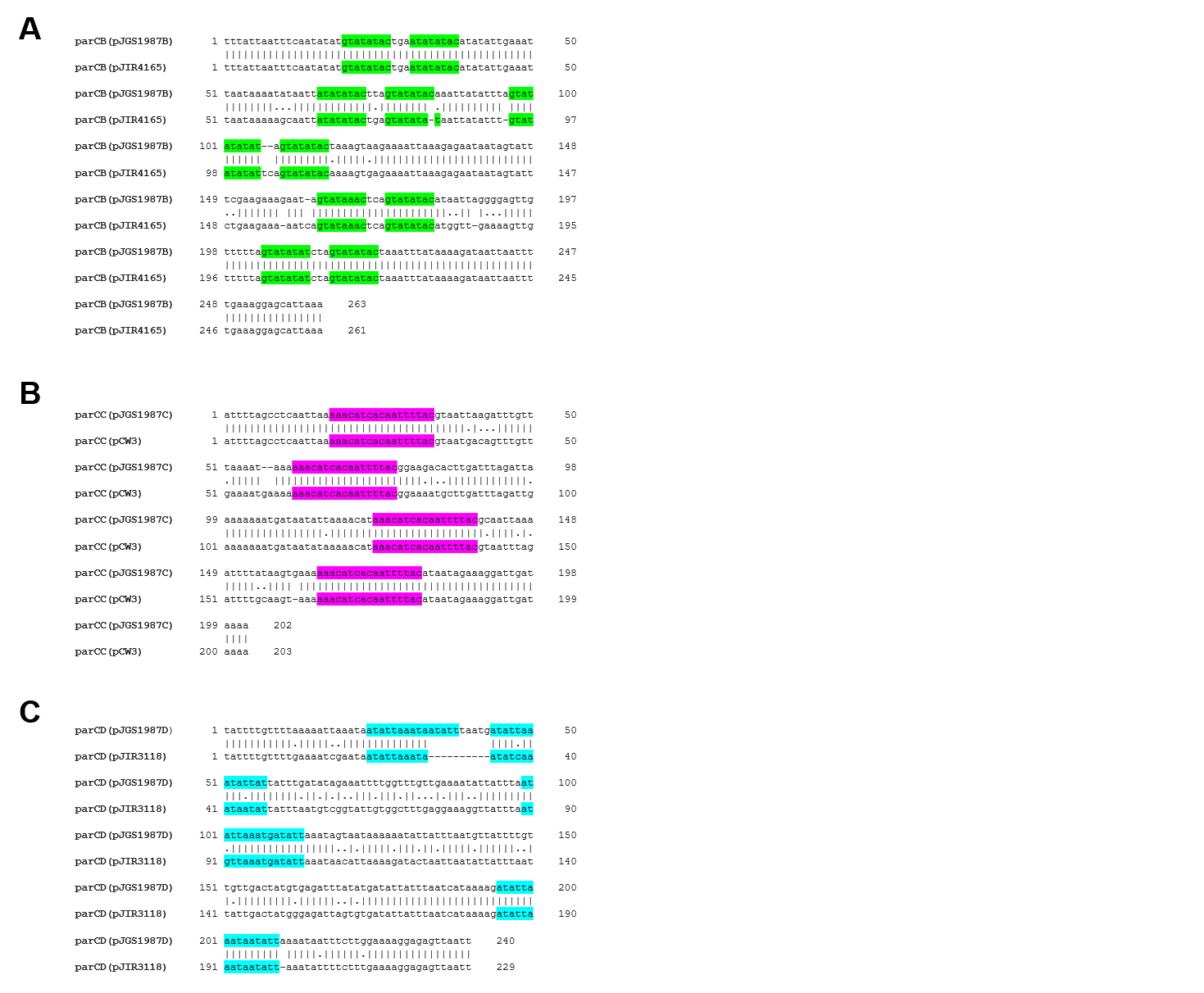

Supplement: FIG S3 [file mbio.01356-22-s0009.docx]
