# Supplementary material for: The Specificity of ParR Binding Determines the Incompatibility of Conjugative Plasmids in Clostridium perfringens
Source: mBio. 2022 Jun 21;13(4):e01356-22. doi: 10.1128/mbio.01356-22 (PMC9426499; doi:10.1128/mbio.01356-22)

**Supplementary Figure 4. Representative SPR binding curves for each ParR homologue and cognate *parC* fragment interactions.** Representative binding curves for each ParR-*parC* pair are shown. **A** shows representative binding curves for ParR_B_ homologues, **B** shows representative binding curves for ParR_C_ homologues, **C** Shows representative binding curves for ParR_D_ homologues. The red curves represent a binding interaction, blue curves represent a non-binding interaction. The origins of the ParR and *parC* fragments are indicated above each graph.
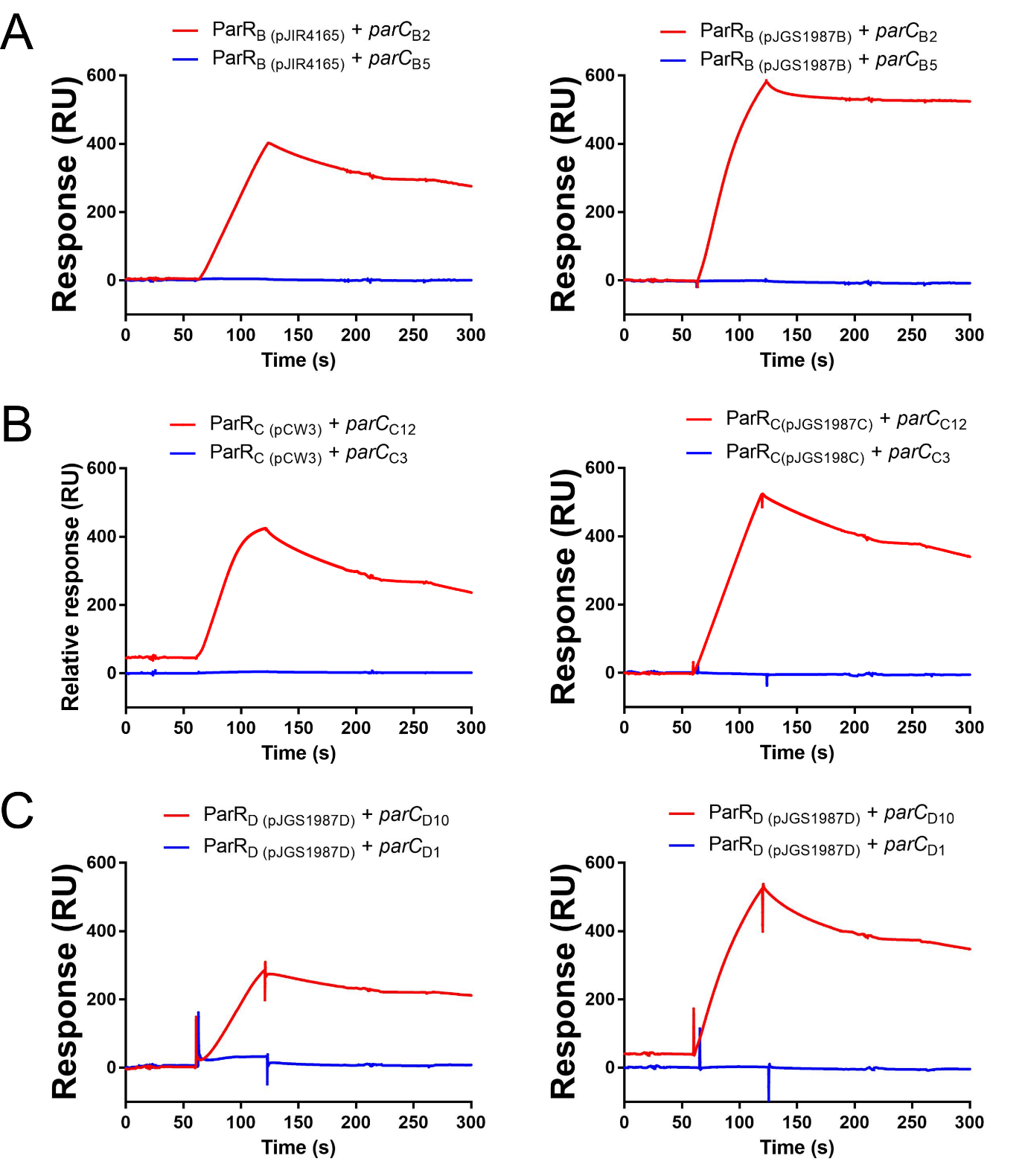

Supplement: FIG S4 [file mbio.01356-22-s0010.docx]
