# Supplementary material for: The Specificity of ParR Binding Determines the Incompatibility of Conjugative Plasmids in Clostridium perfringens
Source: mBio. 2022 Jun 21;13(4):e01356-22. doi: 10.1128/mbio.01356-22 (PMC9426499; doi:10.1128/mbio.01356-22)
Supplement: TABLE S2 [file mbio.01356-22-s0002.docx]

**Supplementary Table 2. ParR proteins properties and expression conditions**

| **Analyte (C-terminal His6 tag)** | **Molecular weight (kDa)** | **Parent plasmid** | ***E. coli* Expression strain** | **Expression media** | **Expression conditions** |  |
| --- | --- | --- | --- | --- | --- | --- |
| ParR_C_  (pCW3) | 10.9 | pCW3 | C43(DE3) | Autoinduction media | 28 °C 24 h/ 22°C 6 h |  |
|  |  |  |  | 2YT + 0.1 mM IPTG | 37 °C 4 h |  |
| ParR_C_  (pJGS1987C) | 11 | pJGS1987C | C43(DE3) | Autoinduction media | 28 °C 24 h/ 22°C 6 h |  |
| ParR_B_  (pJIR4165) | 13.7 | pJIR4165 | C43(DE3) | Autoinduction media | 28 °C 24 h/ 22°C 6 h |  |
|  |  |  |  | 2YT + 0.1 mM IPTG | 37 °C 4 h |  |
| ParR_B_  (pJGS1987B) | 13.7 | pJGS1987B | BL21(DE3) | Autoinduction media | 28 °C 24 h/ 22°C 6 h |  |
| ParR_D_  (pJIR3118) | 12.6 | pJIR3118 | C41(DE3) | Autoinduction media | 28 °C 24 h/ 22°C 6 hrs |  |
|  |  |  |  | 2YT + 0.1 mM IPTG | 37 °C 4 h |  |
| ParR_D_  (pJGS1987D) | 12.2 | pJGS1987D | C41(DE3) | Autoinduction media | 28 °C 24 h/ 22°C 6 h |  |
