# Supplementary material for: The Specificity of ParR Binding Determines the Incompatibility of Conjugative Plasmids in Clostridium perfringens
Source: mBio. 2022 Jun 21;13(4):e01356-22. doi: 10.1128/mbio.01356-22 (PMC9426499; doi:10.1128/mbio.01356-22)
Supplement: TABLE S3 [file mbio.01356-22-s0003.docx]

**Supplementary Table 3. *parC* oligos and ligand molecular weight and size**

| **Oligos** | **Sequence 5’-3’** | **Size (nt)** | **Purpose** |
| --- | --- | --- | --- |
| JRP7138 | 5’-biotinylation-GGATGGGATGCAGGAGGACG | 20 | ReDCaT oligonucleotide |
| JRP6712 | CCTACCCTACGTCCTCCTGC | 20 | ReDCaT complementary (comp) oligonucleotide |
| JRP6917 | TTTATTAATTTCACTATATGTATATACTGA | 30 | B1 |
| JRP6918 | TCAGTATATACATATAGTGAAATTAATAAA^a^ cctaccctacgtcctcctgc^b^ | 50 | B1 comp |
| JRP6919 | TCACTATATGTATATACTGAATATATACAT | 30 | B2 |
| JRP6920 | ATGTATATATTCAGTATATACATATAGTGAcctaccctacgtcctcctgc | 50 | B2 comp |
| JRP6921 | TATATACTGAATATATACATATAGTGTTAT | 30 | B3 |
| JRP6922 | ATAACACTATATGTATATATTCAGTATATAcctaccctacgtcctcctgc | 50 | B3 comp |
| JRP6923 | ATATATACATATAGTGTTATATATAATAAT | 30 | B4 |
| JRP6924 | ATTATTATATATAACACTATATGTATATATcctaccctacgtcctcctgc | 50 | B4 comp |
| JRP6925 | ATAGTGTTATATATAATAATCAATTATATA | 30 | B5 |
| JRP6926 | TATATAATTGATTATTATATATAACACTATcctaccctacgtcctcctgc | 50 | B5 comp |
| JRP6927 | ATATAATAATCAATTATATATACTCAGTAT | 30 | B6 |
| JRP6928 | ATACTGAGTATATATAATTGATTATTATATcctaccctacgtcctcctgc | 50 | B6 comp |
| JRP6929 | CAATTATATATACTCAGTATATATAATTAT | 30 | B7 |
| JRP6930 | ATAATTATATATACTGAGTATATATAATTGcctaccctacgtcctcctgc | 50 | B7 comp |
| JRP6931 | TACTCAGTATATATAATTATATTTTGTATA | 30 | B8 |
| JRP6932 | TATACAAAATATAATTATATATACTGAGTAcctaccctacgtcctcctgc | 50 | B8 comp |
| JRP6933 | ATATAATTATATTTTGTATATATTCAGTAT | 30 | B9 |
| JRP6934 | ATACTGAATATATACAAAATATAATTATATcctaccctacgtcctcctgc | 50 | B9 comp |
| JRP6935 | ATTTTGTATATATTCAGTATATATAAAAAG | 30 | B10 |
| JRP6936 | CTTTTTATATATACTGAATATATACAAAATcctaccctacgtcctcctgc | 50 | B10 comp |
| JRP6937 | TATTCAGTATATATAAAAAGTGAGGGATTT | 30 | B11 |
| JRP6938 | AAATCCCTCACTTTTTATATATACTGAATAcctaccctacgtcctcctgc | 50 | B11 comp |
| JRP6939 | ATATAAAAAGTGAGGGATTTAAAGAGAATA | 30 | B12 |
| JRP6940 | TATTCTCTTTAAATCCCTCACTTTTTATATcctaccctacgtcctcctgc | 50 | B12 comp |
| JRP6941 | TGAGGGATTTAAAGAGAATAATAGTATTTT | 30 | B13 |
| JRP6942 | AAAATACTATTATTCTCTTTAAATCCCTCAcctaccctacgtcctcctgc | 50 | B13 comp |
| JRP6943 | AAAGAGAATAATAGTATTTTGAAGAAAAAT | 30 | B14 |
| JRP6944 | ATTTTTCTTCAAAATACTATTATTCTCTTTcctaccctacgtcctcctgc | 50 | B14 comp |
| JRP6945 | ATAGTATTTTGAAGAAAAATAGGTATAAAC | 30 | B15 |
| JRP6946 | GTTTATACCTATTTTTCTTCAAAATACTATcctaccctacgtcctcctgc | 50 | B15 comp |
| JRP6947 | GAAGAAAAATAGGTATAAACTCAGTATATA | 30 | B16 |
| JRP6948 | TATATACTGAGTTTATACCTATTTTTCTTCcctaccctacgtcctcctgc | 50 | B16 comp |
| JRP6949 | AGGTATAAACTCAGTATATACATGATTGAA | 30 | B17 |
| JRP6950 | TTCAATCATGTATATACTGAGTTTATACCTcctaccctacgtcctcctgc | 50 | B17 comp |
| JRP6951 | TCAGTATATACATGATTGAAAAGTTGTTTT | 30 | B18 |
| JRP6952 | AAAACAACTTTTCAATCATGTATATACTGAcctaccctacgtcctcctgc | 50 | B18 comp |
| JRP6953 | CATGATTGAAAAGTTGTTTTTAGTATATAT | 30 | B19 |
| JRP6954 | ATATATACTAAAAACAACTTTTCAATCATGcctaccctacgtcctcctgc | 50 | B19 comp |
| JRP6955 | AAGTTGTTTTTAGTATATATCTAGTATATA | 30 | B20 |
| JRP6956 | TATATACTAGATATATACTAAAAACAACTTcctaccctacgtcctcctgc | 50 | B20 comp |
| JRP6957 | TAGTATATATCTAGTATATACTAAATTTAT | 30 | B21 |
| JRP6958 | ATAAATTTAGTATATACTAGATATATACTAcctaccctacgtcctcctgc | 50 | B21 comp |
| JRP6959 | CTAGTATATACTAAATTTATAAAAGATAAT | 30 | B22 |
| JRP6960 | ATTATCTTTTATAAATTTAGTATATACTAGcctaccctacgtcctcctgc | 50 | B22 comp |
| JRP6961 | CTAAATTTATAAAAGATAATTAATTTTGAA | 30 | B23 |
| JRP6962 | TTCAAAATTAATTATCTTTTATAAATTTAGcctaccctacgtcctcctgc | 50 | B23 comp |
| JRP6963 | AAAAGATAATTAATTTTGAAAGGAGCATTA | 30 | B24 |
| JRP6964 | TAATGCTCCTTTCAAAATTAATTATCTTTTcctaccctacgtcctcctgc | 50 | B24 comp |
| JRP6965 | AAGATAATTAATTTTGAAAGGAGCATTAAA | 30 | B25 |
| JRP6966 | TTTAATGCTCCTTTCAAAATTAATTATCTTcctaccctacgtcctcctgc | 50 | B25 comp |
| JRP6713 | AATTAAAAACATCACAATTTTACGTAATGA | 30 | C1 |
| JRP6714 | TCATTACGTAAAATTGTGATGTTTTTAATTcctaccctacgtcctcctgc | 50 | C1 comp |
| JRP6715 | ATCACAATTTTACGTAATGACAGTTTGTTG | 30 | C2 |
| JRP6716 | CAACAAACTGTCATTACGTAAAATTGTGATcctaccctacgtcctcctgc | 50 | C2 comp |
| JRP6717 | TACGTAATGACAGTTTGTTGAAAATGAAAA | 30 | C3 |
| JRP6718 | TTTTCATTTTCAACAAACTGTCATTACGTAcctaccctacgtcctcctgc | 50 | C3 comp |
| JRP6719 | CAGTTTGTTGAAAATGAAAAAAACATCACA | 30 | C4 |
| JRP6720 | TGTGATGTTTTTTTCATTTTCAACAAACTGcctaccctacgtcctcctgc | 50 | C4 comp |
| JRP6721 | AAAATGAAAAAAACATCACAATTTTACGGA | 30 | C5 |
| JRP6722 | TCCGTAAAATTGTGATGTTTTTTTCATTTTcctaccctacgtcctcctgc | 50 | C5 comp |
| JRP6723 | AAACATCACAATTTTACGGAAAATGCTTGA | 30 | C6 |
| JRP6724 | TCAAGCATTTTCCGTAAAATTGTGATGTTTcctaccctacgtcctcctgc | 50 | C6 comp |
| JRP6725 | ATTTTACGGAAAATGCTTGATTTAGATTGA | 30 | C7 |
| JRP6726 | TCAATCTAAATCAAGCATTTTCCGTAAAATcctaccctacgtcctcctgc | 50 | C7 comp |
| JRP6727 | AAATGCTTGATTTAGATTGAAAAAAATGAT | 30 | C8 |
| JRP6728 | ATCATTTTTTTCAATCTAAATCAAGCATTTcctaccctacgtcctcctgc | 50 | C8 comp |
| JRP6729 | TTTAGATTGAAAAAAATGATAATATAAAAA | 30 | C9 |
| JRP6730 | TTTTTATATTATCATTTTTTTCAATCTAAAcctaccctacgtcctcctgc | 50 | C9 comp |
| JRP6731 | AAAAAATGATAATATAAAAACATAAACATC | 30 | C10 |
| JRP6732 | GATGTTTATGTTTTTATATTATCATTTTTTcctaccctacgtcctcctgc | 50 | C10 comp |
| JRP6733 | AATATAAAAACATAAACATCACAATTTTAC | 30 | C11 |
| JRP6734 | GTAAAATTGTGATGTTTATGTTTTTATATTcctaccctacgtcctcctgc | 50 | C11 comp |
| JRP6735 | CATAAACATCACAATTTTACGTAATTTAGA | 30 | C12 |
| JRP6736 | TCTAAATTACGTAAAATTGTGATGTTTATGcctaccctacgtcctcctgc | 50 | C12 comp |
| JRP6737 | ACAATTTTACGTAATTTAGATTTTGCAAGT | 30 | C13 |
| JRP6738 | ACTTGCAAAATCTAAATTACGTAAAATTGTcctaccctacgtcctcctgc | 50 | C13 comp |
| JRP6739 | GTAATTTAGATTTTGCAAGTAAAAAACATC | 30 | C14 |
| JRP6740 | GATGTTTTTTACTTGCAAAATCTAAATTACcctaccctacgtcctcctgc | 50 | C14 comp |
| JRP6741 | TTTTGCAAGTAAAAAACATCACAATTTTAC | 30 | C15 |
| JRP6742 | GTAAAATTGTGATGTTTTTTACTTGCAAAAcctaccctacgtcctcctgc | 50 | C15 comp |
| JRP6743 | AAAAAACATCACAATTTTACATAATAGAAA | 30 | C16 |
| JRP6744 | TTTCTATTATGTAAAATTGTGATGTTTTTTcctaccctacgtcctcctgc | 50 | C16 comp |
| JRP6745 | ACAATTTTACATAATAGAAAGGATTGATAA | 30 | C17 |
| JRP6746 | TTATCAATCCTTTCTATTATGTAAAATTGTcctaccctacgtcctcctgc | 50 | C17 comp |
| JRP6747 | AATTTTACATAATAGAAAGGATTGATAAAA | 30 | C18 |
| JRP6748 | TTTTATCAATCCTTTCTATTATGTAAAATTcctaccctacgtcctcctgc | 50 | C18 comp |
| JRP6967 | TTTATTTTGTTTTGAAAATCGAATAATATT | 30 | D1 |
| JRP6968 | AATATTATTCGATTTTCAAAACAAAATAAAcctaccctacgtcctcctgc | 50 | D1 comp |
| JRP6969 | TTTGAAAATCGAATAATATTAAATAATATC | 30 | D2 |
| JRP6970 | GATATTATTTAATATTATTCGATTTTCAAAcctaccctacgtcctcctgc | 50 | D2 comp |
| JRP6971 | GAATAATATTAAATAATATCAAATAATATT | 30 | D3 |
| JRP6972 | AATATTATTTGATATTATTTAATATTATTCcctaccctacgtcctcctgc | 50 | D3 comp |
| JRP6973 | AAATAATATCAAATAATATTATTTAATGTC | 30 | D4 |
| JRP6974 | GACATTAAATAATATTATTTGATATTATTTcctaccctacgtcctcctgc | 50 | D4 comp |
| JRP6975 | AAATAATATTATTTAATGTCGGTATTGTGG | 30 | D5 |
| JRP6976 | CCACAATACCGACATTAAATAATATTATTTcctaccctacgtcctcctgc | 50 | D5 comp |
| JRP6977 | ATTTAATGTCGGTATTGTGGCTTTGAGGAA | 30 | D6 |
| JRP6978 | TTCCTCAAAGCCACAATACCGACATTAAATcctaccctacgtcctcctgc | 50 | D6 comp |
| JRP6979 | GGTATTGTGGCTTTGAGGAAAGGTTATTTA | 30 | D7 |
| JRP6980 | TAAATAACCTTTCCTCAAAGCCACAATACCcctaccctacgtcctcctgc | 50 | D7 comp |
| JRP6981 | CTTTGAGGAAAGGTTATTTAATGTTAAATG | 30 | D8 |
| JRP6982 | CATTTAACATTAAATAACCTTTCCTCAAAGcctaccctacgtcctcctgc | 50 | D8 comp |
| JRP6983 | AGGTTATTTAATGTTAAATGATATTAAATA | 30 | D9 |
| JRP6984 | TATTTAATATCATTTAACATTAAATAACCTcctaccctacgtcctcctgc | 50 | D9 comp |
| JRP6985 | ATGTTAAATGATATTAAATAACATTAAAAG | 30 | D10 |
| JRP6986 | CTTTTAATGTTATTTAATATCATTTAACATcctaccctacgtcctcctgc | 50 | D10 comp |
| JRP6987 | ATATTAAATAACATTAAAAGATACTAATTA | 30 | D11 |
| JRP6988 | TAATTAGTATCTTTTAATGTTATTTAATATcctaccctacgtcctcctgc | 50 | D11 comp |
| JRP6989 | ACATTAAAAGATACTAATTAATATTATTTA | 30 | D12 |
| JRP6990 | TAAATAATATTAATTAGTATCTTTTAATGTcctaccctacgtcctcctgc | 50 | D12 comp |
| JRP6991 | ATACTAATTAATATTATTTAATTATTGACT | 30 | D13 |
| JRP6992 | AGTCAATAATTAAATAATATTAATTAGTATcctaccctacgtcctcctgc | 50 | D13 comp |
| JRP6993 | ATATTATTTAATTATTGACTATGGGAGATT | 30 | D14 |
| JRP6994 | AATCTCCCATAGTCAATAATTAAATAATATcctaccctacgtcctcctgc | 50 | D14 comp |
| JRP6995 | ATTATTGACTATGGGAGATTAGTGTGATAT | 30 | D15 |
| JRP6996 | ATATCACACTAATCTCCCATAGTCAATAATcctaccctacgtcctcctgc | 50 | D15 comp |
| JRP6997 | ATGGGAGATTAGTGTGATATTATTTAATCA | 30 | D16 |
| JRP6998 | TGATTAAATAATATCACACTAATCTCCCATcctaccctacgtcctcctgc | 50 | D16 comp |
| JRP6999 | AGTGTGATATTATTTAATCATAAAAGATAT | 30 | D17 |
| JRP7000 | ATATCTTTTATGATTAAATAATATCACACTcctaccctacgtcctcctgc | 50 | D17 comp |
| JRP7001 | TATTTAATCATAAAAGATATTAAATAATAT | 30 | D18 |
| JRP7002 | ATATTATTTAATATCTTTTATGATTAAATAcctaccctacgtcctcctgc | 50 | D18 comp |
| JRP7003 | TAAAAGATATTAAATAATATTAAATATTTT | 30 | D19 |
| JRP7004 | AAAATATTTAATATTATTTAATATCTTTTAcctaccctacgtcctcctgc | 50 | D19 comp |
| JRP7005 | TAAATAATATTAAATATTTTCTTTGAAAAG | 30 | D20 |
| JRP7006 | CTTTTCAAAGAAAATATTTAATATTATTTAcctaccctacgtcctcctgc | 50 | D20 comp |
| JRP7007 | TAAATATTTTCTTTGAAAAGGAGAGTTAAT | 30 | D21 |
| JRP7008 | ATTAACTCTCCTTTTCAAAGAAAATATTTAcctaccctacgtcctcctgc | 50 | D21 comp |
| JRP7256 | AATTAAAAAAAAAACAATTTTACGTAATGA | 30 | C1-5’F |
| JRP7257 | TCATTACGTAAAATTGTTTTTTTTTTAATTcctaccctacgtcctcctgc | 50 | C1-5’R |
| JRP7258 | AATTAAAAACATCAAAAAAAAAAGTAATGA | 30 | C1-3’F |
| JRP7259 | TCATTACTTTTTTTTTTGATGTTTTTAATTcctaccctacgtcctcctgc | 50 | C1-3’R |
| JRP7260 | AATTAAAAAAAAAAAAAAAAAAAGTAATGA | 30 | C1deltaF |
| JRP7261 | TCATTACTTTTTTTTTTTTTTTTTTTAATTcctaccctacgtcctcctgc | 50 | C1deltaR |
| JRP7262 | AGAAAAAAAATCAGTATATACATGATTGAA | 30 | B17-5’F |
| JRP7263 | TTCAATCATGTATATACTGATTTTTTTTCTcctaccctacgtcctcctgc | 50 | B17-5’R |
| JRP7264 | AGGTATAAACTCAAAAAAAAAATGATTGAA | 30 | B17-3’F |
| JRP7265 | TTCAATCATTTTTTTTTTGAGTTTATACCTcctaccctacgtcctcctgc | 50 | B17-3’R |
| JRP7266 | AGAAAAAAAATCAAAAAAAAAATGATTGAA | 30 | B17-delta’F |
| JRP7267 | TTCAATCATTTTTTTTTTGATTTTTTTTCTcctaccctacgtcctcctgc | 50 | B17-delta’R |
| JRP7268 | AGGTATAAACGTATATACATGATTGAA | 30 | B17-delta middleF |
| JRP7269 | TTCAATCATGTATATACGTTTATACCTcctaccctacgtcctcctgc | 50 | B17-delta middleR |
| JRP7270 | GAATAATATTAAATAATATCAAATAATATTATTTAATGTC |  | *parC_D_* 3+4F |
| JRP7271 | GACATTAAATAATATTATTTGATATTATTTAATATTATTCcctaccctacgtcctcctgc |  | *parC_D_* 3+4R |
| JRP7272 | ATAATATTAAATAATATCAAATAATATTATTTAATGTC |  | *parC_D_* LHS1F |
| JRP7273 | GACATTAAATAATATTATTTGATATTATTTAATATTATcctaccctacgtcctcctgc |  | *parC_D_* LHS1R |
| JRP7274 | AATATTAAATAATATCAAATAATATTATTTAATGTC |  | *parC_D_* LHS2F |
| JRP7275 | GACATTAAATAATATTATTTGATATTATTTAATATTcctaccctacgtcctcctgc |  | *parC_D_* LHS2R |
| JRP7276 | TATTAAATAATATCAAATAATATTATTTAATGTC |  | *parC_D_* LHS3F |
| JRP7277 | GACATTAAATAATATTATTTGATATTATTTAATAcctaccctacgtcctcctgc |  | *parC_D_* LHS3R |
| JRP7278 | TTAAATAATATCAAATAATATTATTTAATGTC |  | *parC_D_* LHS4F |
| JRP7279 | GACATTAAATAATATTATTTGATATTATTTAAcctaccctacgtcctcctgc |  | *parC_D_* LHS4R |
| JRP7280 | AAATAATATCAAATAATATTATTTAATGTC |  | *parC_D_* LHS5F |
| JRP7281 | GACATTAAATAATATTATTTGATATTATTTcctaccctacgtcctcctgc |  | *parC_D_* LHS5R |
| JRP7282 | ATAATATCAAATAATATTATTTAATGTC |  | *parC_D_* LHS6F |
| JRP7283 | GACATTAAATAATATTATTTGATATTATcctaccctacgtcctcctgc |  | *parC_D_* LHS6R |
| JRP7284 | AATATCAAATAATATTATTTAATGTC |  | *parC_D_* LHS7F |
| JRP7285 | GACATTAAATAATATTATTTGATATTcctaccctacgtcctcctgc |  | *parC_D_* LHS7R |
| JRP7286 | TATCAAATAATATTATTTAATGTC |  | *parC_D_* LHS8F |
| JRP7287 | GACATTAAATAATATTATTTGATAcctaccctacgtcctcctgc |  | *parC_D_* LHS8R |
| JRP7288 | TCAAATAATATTATTTAATGTC |  | *parC_D_* LHS9F |
| JRP7289 | GACATTAAATAATATTATTTGAcctaccctacgtcctcctgc |  | *parC_D_* LHS9R |
| JRP7290 | AAATAATATTATTTAATGTC |  | *parC_D_* LHS10F |
| JRP7291 | GACATTAAATAATATTATTTcctaccctacgtcctcctgc |  | *parC_D_* LHS10R |
| JRP7292 | ATAATATTATTTAATGTC |  | *parC_D_* LHS11F |
| JRP7293 | GACATTAAATAATATTATcctaccctacgtcctcctgc |  | *parC_D_* LHS11R |
| JRP7294 | AATATTATTTAATGTC |  | *parC_D_* LHS12F |
| JRP7295 | GACATTAAATAATATTcctaccctacgtcctcctgc |  | *parC_D_* LHS12R |
| JRP7296 | GAATAATATTAAATAATATCAAATAATATTATTTAATG |  | *parC_D_* RHS1F |
| JRP7297 | CATTAAATAATATTATTTGATATTATTTAATATTATTCcctaccctacgtcctcctgc |  | *parC_D_* RHS1R |
| JRP7298 | GAATAATATTAAATAATATCAAATAATATTATTTAA |  | *parC_D_* RHS2F |
| JRP7299 | TTAAATAATATTATTTGATATTATTTAATATTATTCcctaccctacgtcctcctgc |  | *parC_D_* RHS2R |
| JRP7300 | GAATAATATTAAATAATATCAAATAATATTATTT |  | *parC_D_* RHS3F |
| JRP7301 | AAATAATATTATTTGATATTATTTAATATTATTCcctaccctacgtcctcctgc |  | *parC_D_* RHS3R |
| JRP7302 | GAATAATATTAAATAATATCAAATAATATTAT |  | *parC_D_* RHS4F |
| JRP7303 | ATAATATTATTTGATATTATTTAATATTATTCcctaccctacgtcctcctgc |  | *parC_D_* RHS4R |
| JRP7304 | GAATAATATTAAATAATATCAAATAATATT |  | *parC_D_* RHS5F |
| JRP7305 | AATATTATTTGATATTATTTAATATTATTCcctaccctacgtcctcctgc |  | *parC_D_* RHS5R |
| JRP7306 | GAATAATATTAAATAATATCAAATAATA |  | *parC_D_* RHS6F |
| JRP7307 | TATTATTTGATATTATTTAATATTATTCcctaccctacgtcctcctgc |  | *parC_D_* RHS6R |
| JRP7308 | GAATAATATTAAATAATATCAAATAA |  | *parC_D_* RHS7F |
| JRP7309 | TTATTTGATATTATTTAATATTATTCcctaccctacgtcctcctgc |  | *parC_D_* RHS7R |
| JRP7310 | GAATAATATTAAATAATATCAAAT |  | *parC_D_* RHS8F |
| JRP7311 | ATTTGATATTATTTAATATTATTCcctaccctacgtcctcctgc |  | *parC_D_* RHS8R |
| JRP7312 | GAATAATATTAAATAATATCAA |  | *parC_D_* RHS9F |
| JRP7313 | TTGATATTATTTAATATTATTCcctaccctacgtcctcctgc |  | *parC_D_* RHS9R |
| JRP7314 | GAATAATATTAAATAATATC |  | *parC_D_* RHS10F |
| JRP7315 | GATATTATTTAATATTATTCcctaccctacgtcctcctgc |  | *parC_D_* RHS10R |
| JRP7316 | GAATAATATTAAATAATA |  | *parC_D_* RHS11F |
| JRP7317 | TATTATTTAATATTATTCcctaccctacgtcctcctgc |  | *parC_D_* RHS11R |
| JRP7318 | GAATAATATTAAATAA |  | *parC_D_* RHS12F |
| JRP7319 | TTATTTAATATTATTCcctaccctacgtcctcctgc |  | *parC_D_* RHS12R |

^a^Uppercase sequence corresponds to the unique *parC* fragment

^b^Lowercase sequence corresponds to the 3’ ReDCaT complementary tail
