# Supplementary material for: The Specificity of ParR Binding Determines the Incompatibility of Conjugative Plasmids in Clostridium perfringens
Source: mBio. 2022 Jun 21;13(4):e01356-22. doi: 10.1128/mbio.01356-22 (PMC9426499; doi:10.1128/mbio.01356-22)
Supplement: TABLE S4 [file mbio.01356-22-s0004.docx]

**Supplementary Table 4. Binding of ParR homologues to *parC* using surface plasmon resonance**

|  | **ParR_B_(pJIR4165)** | | | | **ParR_C_(pCW3)** | | | | **ParR_D_(pJIR3118)** | | | |
| --- | --- | --- | --- | --- | --- | --- | --- | --- | --- | --- | --- | --- |
|  | Replicate 1 | | Replicate 2 | | Replicate 1 | | Replicate 2^#^ | | Replicate 1 | | Replicate 2 | |
| Oligo | DNA Captured (RU) | Response (RU) | DNA Captured (RU) | Response (RU) | DNA captured (RU) | Response (RU) | DNA captured (RU) | Response (RU) replicate 2 | DNA captured (RU) | Response (RU) | DNA Captured (RU) | Response (RU) |
| B1 | 210.3 | 58 | 162.1 | 10.1 | 313.1 | -2.8 |  |  | 251.5 | 1.1 |  |  |
| B2 | 266.2 | 383.7 | 169.3 | 88.7 | 345 | -2.7 |  |  | 264.9 | 2.3 |  |  |
| B3 | 257.6 | 133.8 | 164.3 | 26.3 | 366.2 | -3 |  |  | 256.8 | 1.3 |  |  |
| B4 | 258 | 2.1 | 171.1 | -1.8 | 352.9 | -3.4 |  |  | 258.8 | 1.6 |  |  |
| B5 | 261.5 | 1.1 | 174.6 | -2.4 | 348.1 | -2.9 |  |  | 251.4 | 0.6 |  |  |
| B6 | 239.1 | 166.9 | 173.9 | 61.5 | 355.8 | -3.3 |  |  | 258.4 | 1.5 |  |  |
| B7 | 199.6 | 52.2 | 132.5 | 12.3 | 300.4 | -3.1 |  |  | 206.1 | 0.3 |  |  |
| B8 | 266.7 | 69.6 | 182.2 | 11.1 | 351 | -3 |  |  | 270.5 | 0.1 |  |  |
| B9 | 264.1 | 118.9 | 184.9 | 33.1 | 360.4 | -3.1 |  |  | 263 | 2.1 |  |  |
| B10 | 163 | 216.4 | 190.4 | 61.1 | 379.3 | -3 |  |  | 268.4 | 0 |  |  |
| B11 | 242.5 | 4.2 | 168.7 | -1.9 | 370.8 | -3.3 |  |  | 246.6 | -0.7 |  |  |
| B12 | 264.4 | 0.7 | 182.6 | -2.5 | 371.2 | -3.6 |  |  | 261.3 | -0.9 |  |  |
| B13 | 239 | 0.7 | 201.2 | -2.8 | 313.9 | -2.8 |  |  | 203.9 | -0.6 |  |  |
| B14 | 291.1 | 0.3 | 206.9 | -2.5 | 364.6 | -3 |  |  | 287.3 | 1.8 |  |  |
| B15 | 275.5 | 0.4 | 191.2 | -2.4 | 398.3 | -3.3 |  |  | 272.1 | 0.2 |  |  |
| B16 | 279.3 | 2.3 | 204.4 | -3 | 378.8 | -3.6 |  |  | 273.9 | -2 |  |  |
| B17 | 274.9 | 368.6 | 202.3 | 77.1 | 366.8 | -2.8 |  |  | 275.8 | -1.2 |  |  |
| B18 | 265.2 | 99.8 | 204.5 | 21.2 | 369 | -2.9 |  |  | 267.9 | -0.6 |  |  |
| B19 | 288.4 | 2.4 | 213.8 | -2.6 | 391.7 | -3.4 |  |  | 284.8 | 5.5 |  |  |
| B20 | 205.8 | 10.1 | 217.6 | -1.6 | 381.7 | -3.4 |  |  | 277 | 1.5 |  |  |
| B21 | 235.6 | 377.2 | 195.3 | 83.2 | 318.8 | -3.4 |  |  | 248.4 | 2.4 |  |  |
| B22 | 248.3 | 156.5 | 210 | 38.6 | 394.8 | -2.8 |  |  | 273.1 | 2.6 |  |  |
| B23 | 149.9 | 3.2 | 220.8 | -2.8 | 387.4 | -4.1 |  |  | 279.4 | 1.2 |  |  |
| B24 | 287.3 | 2 | 216.9 | -2.9 | 393.8 | -3.4 |  |  | 283.2 | 0.7 |  |  |
| B25 | 256 | 2.2 | 224.6 | -2.9 | 404.1 | -3.6 |  |  | 308.1 | 0.1 |  |  |
| C1 | 237.5 | -1.7 | 191.6 | -3 | 119.5 | 256.3 | 205.1 | 422.1 | 250.9 | 9.7 |  |  |
| C2 | 216.8 | -0.6 | 189.6 | -2.6 | 141.2 | 54.7 | 207.5 | 161.2 | 284.9 | 10.2 |  |  |
| C3 | 209.4 | -1.4 | 192.9 | -3 | 142 | 0.8 | 245.4 | -1.8 | 242.6 | 8.5 |  |  |
| C4 | 224.6 | -2.2 | 188.9 | -2.9 | 140.7 | 0.9 | 223.9 | -1.8 | 282 | 5.6 |  |  |
| C5 | 88.9 | -0.4 | 201.8 | -3.1 | 123.7 | 249.5 | 245.7 | 405.9 | 277.1 | 8.4 |  |  |
| C6 | 206.2 | -1.6 | 207.1 | -3.2 | 149.1 | 282.2 | 244.7 | 427 | 241 | 6.4 |  |  |
| C7 | 253.6 | -1.6 | 219.2 | -3.2 | 141.1 | 9.2 | 246.2 | 34.4 | 306.8 | 6.4 |  |  |
| C8 | 109.1 | -0.5 | 211.9 | -3.3 | 160.3 | 1.5 | 259.6 | 1.7 | 314.5 | 7 |  |  |
| C9 | 155.1 | -0.9 | 206.4 | -3.3 | 166.2 | 0.4 | 232.3 | -1.5 | 272.6 | 8.1 |  |  |
| C10 | 232.9 | -1.4 | 220.9 | -3.3 | 162.6 | 1.5 | 262.6 | -1 | 271.3 | 14.8 |  |  |
| C11 | 223 | -1.7 | 220.6 | -3.7 | 148.7 | 154.7 | 266.8 | 311.3 | 300.2 | 12.4 |  |  |
| C12 | 241.9 | -1.7 | 220.5 | -3.6 | 187.8 | 348.4 | 267.7 | 448.4 | 286.2 | 8.5 |  |  |
| C13 | 264.3 | -2.1 | 230.5 | -3.8 | 159 | 48.4 | 279.4 | 173.7 | 280.8 | 6.6 |  |  |
| C14 | 254.3 | -3.1 | 222.4 | -3.6 | 161.8 | 42.6 | 371.1 | 101.9 | 339.8 | 7.1 |  |  |
| C15 | 266.1 | -2 | 233.2 | -3.3 | 169 | 217 | 437.9 | 401.3 | 516.4 | 17.4 |  |  |
| C16 | 267.1 | -2.6 | 236 | -3.4 | 157.1 | 311.2 | 302.2 | 435 | 419.1 | 13.5 |  |  |
| C17 | 230.6 | -0.9 | 223.5 | -3.1 | 168.9 | 4.3 | 408.2 | 9.3 | 329.8 | 5.7 |  |  |
| C18 | 259.8 | -2.1 | 232.9 | -3.5 | 182.8 | 3.1 | 288 | 4 | 334.2 | 5.9 |  |  |
| D1 | 218.2 | -3.8 | 158.9 | -6 | 225.8 | -5.1 |  |  | 111.8 | 7 | 236.8 | 5.4 |
| D2 | 223.9 | -2.6 | 167.6 | -5.7 | 228 | -4.6 |  |  | 114.7 | 36.4 | 243 | 37.9 |
| D3 | 217.7 | -3.8 | 160.3 | -6.2 | 233.3 | -4.9 |  |  | 111.8 | 225 | 235.6 | 155.1 |
| D4 | 185 | -2.9 | 164.6 | -5.7 | 232.2 | -4.8 |  |  | 109.7 | 232.7 | 240.8 | 157.8 |
| D5 | 214.3 | -3.9 | 168.2 | -6.2 | 229.9 | -4 |  |  | 113.4 | 80.5 | 251.1 | 75.5 |
| D6 | 215.5 | -2.9 | 169.7 | -5.4 | 239.2 | -3.4 |  |  | 116.1 | 8.9 | 260 | 4.3 |
| D7 | 200.3 | -3.2 | 168.9 | -5.7 | 255.6 | -4.6 |  |  | 114.3 | 8.4 | 256.4 | 3.9 |
| D8 | 191.1 | -2.4 | 181.4 | -5.8 | 249 | -4.8 |  |  | 121.7 | 15.5 | 276 | 10.3 |
| D9 | 85.4 | -0.4 | 178.3 | -6.1 | 273.5 | -4.6 |  |  | 111.3 | 213.4 | 254.3 | 149.7 |
| D10 | 103.6 | -0.7 | 188.4 | -6 | 246.3 | -4.7 |  |  | 111.8 | 270.1 | 261.6 | 169.8 |
| D11 | 166.1 | -3.7 | 191.3 | -6.8 | 258 | -4.4 |  |  | 115.1 | 236.5 | 273.3 | 157.6 |
| D12 | 226.1 | -3.2 | 189.4 | -6.1 | 270.5 | -4.2 |  |  | 115.9 | 250 | 277 | 158.8 |
| D13 | 242.3 | -3.9 | 197.7 | -6.7 | 273.4 | -4.3 |  |  | 110.2 | 187.9 | 272.3 | 138.2 |
| D14 | 238.5 | -4.1 | 196.7 | -7 | 266.5 | -4.8 |  |  | 112.3 | 28.9 | 279.9 | 27.7 |
| D15 | 204.1 | -2.6 | 207.7 | -6.1 | 273.4 | -4.2 |  |  | 113.8 | 9.6 | 289.3 | 3.5 |
| D16 | 140.5 | -1.4 | 212 | -6.2 | 282.4 | -4.1 |  |  | 114.9 | 21.5 | 289.7 | 25.8 |
| D17 | 219.8 | -3.4 | 198.7 | -6.6 | 287.6 | -4 |  |  | 112.2 | 92.2 | 279.7 | 91.4 |
| D18 | 228.3 | -3.8 | 194.9 | -6.3 | 278.4 | -4.3 |  |  | 109.5 | 68 | 283.1 | 60.5 |
| D19 | 235.7 | -3.7 | 190.3 | -5.6 | 280.3 | -4.2 |  |  | 113.6 | 207.1 | 291.9 | 145 |
| D20 | 245.5 | -4.1 | 211 | -6.6 | 298.8 | -4.4 |  |  | 114.3 | 111.4 | 297.1 | 97.7 |
| D21 | 217.3 | -3.6 | 215.5 | -7.9 | 295 | -4.3 |  |  | 140.1 | 158.9 | 300.9 | 5.4 |
|  | | | | | | | | | | | | |
|  | **ParR_B_(pJGS1987B)** | | | | **ParR_C_(pJGS1987C)** | | | | **ParR_D_(pJGS1987D)** | | | |
|  | Replicate 1 | | Replicate 2 | | Replicate 1 | | Replicate 2 | | Replicate 1 | | Replicate 2 | |
| Oligo | DNAcaptured (RU) | Response (RU) replicate 1 | DNAcaptured (RU) | Response (RU) replicate 2 | DNAcaptured (RU) | Response (RU) replicate 1 | DNAcaptured (RU) | Response (RU) replicate 2 | DNAcaptured (RU) | Response (RU) replicate 1 | DNAcaptured (RU) | Response (RU) replicate 2 |
| B1 | 211.7 | 260.3 | 107.7 | 25 | 217.6 | -3.1 |  |  | 200.3 | -11.9 |  |  |
| B2 | 233.1 | 557.1 | 128.1 | 158.7 | 72.5 | -5.9 |  |  | 229.5 | -5.1 |  |  |
| B3 | 206.9 | 323.6 | 118.3 | 51.7 | 241.2 | -5.9 |  |  | 223.5 | -6.7 |  |  |
| B4 | 224.5 | 25.3 | 125 | -55.1 | 234.1 | -5.7 |  |  | 223.9 | -3.3 |  |  |
| B5 | 229.5 | -1.3 | 121.9 | -62.2 | 236 | -5.8 |  |  | 227.6 | -6.5 |  |  |
| B6 | 227 | 278 | 121.3 | 45.8 | 241.2 | -5.3 |  |  | 222.3 | -4.3 |  |  |
| B7 | 175.1 | 96 | 92.8 | -47.6 | 237.7 | -5.3 |  |  | 169.3 | -9.3 |  |  |
| B8 | 230.4 | 289 | 144.8 | 37.2 | 187.6 | -6 |  |  | 227 | -5 |  |  |
| B9 | 231.1 | 212.8 | 140.9 | 15.1 | 246.1 | -5 |  |  | 224 | 0.4 |  |  |
| B10 | 233.1 | 483.7 | 106.1 | 100.2 | 243.5 | -5.2 |  |  | 226.6 | -3.9 |  |  |
| B11 | 207 | 102.4 | 113.7 | -26.5 | 244.4 | -5.4 |  |  | 201.2 | -8.5 |  |  |
| B12 | 229.5 | -9 | 106.5 | -61.6 | 223.3 | -5.4 |  |  | 219.8 | -8.6 |  |  |
| B13 | 212.4 | -10 | 144.2 | -61.4 | 237.6 | -5.9 |  |  | 214.3 | -9.3 |  |  |
| B14 | 245.6 | -9 | 88.3 | -58.5 | 237.9 | -5 |  |  | 237.1 | 3.1 |  |  |
| B15 | 225.2 | -8.9 | 110.1 | -58.3 | 262.5 | -5.4 |  |  | 223.7 | -1.2 |  |  |
| B16 | 231.6 | 42.5 | 129.1 | -41.4 | 247.2 | -5.5 |  |  | 228.6 | -7.2 |  |  |
| B17 | 229.7 | 523.7 | 155.9 | 115.6 | 251 | -5.3 |  |  | 225 | -4.9 |  |  |
| B18 | 228.2 | 358.9 | 113.2 | 61.5 | 252.8 | -5.1 |  |  | 227.2 | -4.8 |  |  |
| B19 | 238.2 | -8.1 | 126.1 | -55.3 | 248.2 | -3.1 |  |  | 192.2 | 1.3 |  |  |
| B20 | 211.7 | 260.3 | 127.4 | -3.1 | 274.6 | -4 |  |  | 238.6 | -1 |  |  |
| B21 | 233.1 | 557.1 | 116.5 | 141.7 | 255.2 | -3.8 |  |  | 217.1 | -1.1 |  |  |
| B22 | 206.9 | 323.6 | 126.4 | 86 | 236.1 | -3.3 |  |  | 232.1 | -0.3 |  |  |
| B23 | 224.5 | 25.3 | 101 | -52.9 | 249.6 | -4.3 |  |  | 241.8 | -3.1 |  |  |
| B24 | 229.5 | -1.3 | 110.1 | -53.8 | 260.2 | -3.7 |  |  | 240.3 | -4 |  |  |
| B25 | 227 | 278 | 102.9 | -52.1 | 231.1 | -5 |  |  | 250.1 | -3.6 |  |  |
| C1 | 346.2 | -1.7 |  |  | 333.7 | 511.4 | 148.9 | 64.9 | 339.4 | -1.5 |  |  |
| C2 | 348.2 | 0.1 |  |  | 336.6 | 110.7 | 138.9 | -15.5 | 343 | 14.7 |  |  |
| C3 | 344.1 | 0.3 |  |  | 323.5 | -6.2 | 146.6 | -6.8 | 344.7 | 12.8 |  |  |
| C4 | 323.1 | -0.8 |  |  | 342.2 | -7 | 147.3 | 186.1 | 335 | 10.8 |  |  |
| C5 | 365.1 | 0.3 |  |  | 351.7 | 456.5 | 141.5 | 183.2 | 354.3 | 8.6 |  |  |
| C6 | 322.7 | 4.5 |  |  | 349.8 | 452.6 | 149.7 | 3 | 362.9 | 12.7 |  |  |
| C7 | 1 | -0.2 |  |  | 357.6 | 5.1 | 148.4 | -13.4 | 366.1 | 10.5 |  |  |
| C8 | 367.6 | -2.3 |  |  | 351.6 | -3.5 | 154.6 | -15.6 | 372.7 | 8.2 |  |  |
| C9 | 319.2 | 1.7 |  |  | 360.9 | -5.8 | 147.5 | -14.8 | 369.5 | 6.9 |  |  |
| C10 | 373.4 | 1.9 |  |  | 376 | -5.7 | 148.2 | 96.4 | 381 | 10.5 |  |  |
| C11 | 360.4 | 1.4 |  |  | 364.1 | 343.5 | 150.5 | 195.7 | 397.3 | 20.3 |  |  |
| C12 | 384.2 | 1.1 |  |  | 380.3 | 505.6 | 152.2 | 58.9 | 384 | 12.9 |  |  |
| C13 | 387 | 0.2 |  |  | 379 | 96.6 | 150.5 | 118.4 | 395.7 | 11 |  |  |
| C14 | 395.3 | -1.3 |  |  | 375.5 | 105.4 | 149.9 | 198.8 | 400.5 | 8.6 |  |  |
| C15 | 392.3 | -0.6 |  |  | 386.7 | 428.5 | 152.3 | -8.4 | 400.9 | 7.1 |  |  |
| C16 | 403.8 | 2.6 |  |  | 383.4 | 473.2 | 146 | -11.6 | 409.8 | 11.1 |  |  |
| C17 | 381.5 | -0.4 |  |  | 388.9 | -0.5 | 148.9 | 64.9 | 408.6 | 10.2 |  |  |
| C18 | 389.9 | -0.7 |  |  | 400.6 | -3.8 | 138.9 | -15.5 | 416.6 | 6.9 |  |  |
| D1 | 200.8 | -3.4 |  |  | 326.2 | -11.5 |  |  | 331.6 | -1.3 | 160.5 | -47 |
| D2 | 203.5 | -2.6 |  |  | 322.3 | -11.5 |  |  | 335.2 | 42.8 | 159.8 | 101.1 |
| D3 | 195.1 | -2.3 |  |  | 316.6 | -11.6 |  |  | 332.1 | 341.5 | 152.1 | 111.1 |
| D4 | 200.3 | -1.3 |  |  | 317.9 | -12.6 |  |  | 326.7 | 356.4 | 171.8 | -37 |
| D5 | 211.1 | -1.8 |  |  | 329.3 | -10.1 |  |  | 326.7 | 80.6 | 163.8 | -46.9 |
| D6 | 221.4 | -3.1 |  |  | 339.9 | -13.1 |  |  | 342.2 | -0.7 | 158.6 | -51.4 |
| D7 | 212.6 | -4.3 |  |  | 345.4 | -13 |  |  | 352 | -1.9 | 160.9 | -50.5 |
| D8 | 238.1 | -3.4 |  |  | 348.9 | -10.8 |  |  | 360.5 | 4.8 | 154 | 49.4 |
| D9 | 212.7 | -2.8 |  |  | 336.8 | -11.2 |  |  | 368 | 235.5 | 173.7 | 164.8 |
| D10 | 213.9 | -1.8 |  |  | 351.7 | -11.8 |  |  | 348.2 | 459.8 | 163 | 80.8 |
| D11 | 231.9 | -1.9 |  |  | 354.2 | -11.4 |  |  | 366.6 | 279.6 | 161.7 | 122.4 |
| D12 | 246.6 | -2 |  |  | 365.5 | -11.5 |  |  | 369.4 | 346.7 | 150.9 | 10.3 |
| D13 | 229.4 | -1.8 |  |  | 374.9 | -13 |  |  | 384.6 | 175.4 | 161.6 | -51.6 |
| D14 | 236.5 | -2.9 |  |  | 360.1 | -11.9 |  |  | 395.1 | 21.1 | 164.2 | -45.3 |
| D15 | 241.4 | -3.6 |  |  | 373.8 | -11.4 |  |  | 387 | -2.7 | 163.5 | -31.3 |
| D16 | 241.7 | -3.1 |  |  | 382.2 | -11.1 |  |  | 396.6 | 14.1 | 157.7 | -41.4 |
| D17 | 230.2 | -2.1 |  |  | 376.6 | -11.1 |  |  | 404.2 | 87 | 155.3 | 51.6 |
| D18 | 219.2 | -2.7 |  |  | 385.3 | -11.7 |  |  | 400.3 | 43.8 | 171.2 | -36.7 |
| D19 | 243 | -3 |  |  | 394.1 | -11.6 |  |  | 408.6 | 239.8 | 158.2 | -56.6 |
| D20 | 262.2 | -2.3 |  |  | 396.3 | -11.2 |  |  | 422.4 | 84.8 | 160.5 | -47 |
| D21 | 246.8 | -3.7 |  |  | 399.8 | -11.8 |  |  | 420.5 | -1.3 | 159.8 | 101.1 |

^#^ duplicates were not performed for non-cognate *parC* fragment arrays as, distinct ParR proteins from the same family [e.g. ParR_C_(pCW3) and ParR_C_(pJGS1987)] did not bind to non-cognate DNA fragment arrays, and internal ‘negative controls’ (non-interacting fragments within the cognate *parC* DNA arrays) were consistent between duplicates.
