# Supplementary material for: The Specificity of ParR Binding Determines the Incompatibility of Conjugative Plasmids in Clostridium perfringens
Source: mBio. 2022 Jun 21;13(4):e01356-22. doi: 10.1128/mbio.01356-22 (PMC9426499; doi:10.1128/mbio.01356-22)
Supplement: TABLE S5 [file mbio.01356-22-s0005.docx]

**Supplementary Table 5. Hydrodynamic properties of ParR_C_(pCW3) and *parC_C_*(pCW3) C5 DNA determined by AUC experiments**

| **Components** | **Mr^a^**  **kDa** | **MW^b^ (Da)** | **Sedimentation coefficient (*s*)^c^** | **Standardised sedimentation coefficient (*s*_20,W_)** | **Oligomeric state** | **Partial specific volume** |
| --- | --- | --- | --- | --- | --- | --- |
| **ParR_C_(pCW3)** | 10.9 | 48439 | 2.86 | 3.1 | Tetramer | 0.7372 |
| ***parC*_C_ (pCW3) C5** | 18.6 | 18426 | 2.6 | 2.8 | Double stranded DNA | 0.55 |
| ***parC_C_* (pCW3) C9** | 18.6 | 18426 |  |  | Double stranded DNA | 0.55 |

^a^ Relative molecular weight calculated from amino acid sequence.

^b^ Apparent molecular weight derived from c(M) analysis

^c^ Sedimentation coefficient taken from the ordinate maximum of c(s) distribution
